# Cross-Priming and Cross-Tolerance After Intramuscular mRNA Vaccination for Viral Infections: Feasibility and Implications

**DOI:** 10.3390/life15101575

**Published:** 2025-10-09

**Authors:** Siguna Mueller

**Affiliations:** Center for Research in Medical Pharmacology, University of Insubria, 21100 Varese, Italy; smuellermueller@uninsubria.it

**Keywords:** mRNA vaccines, CD8+ T cell response, antigen cross-presentation, pro-drug, systemic effects, tolerance, specificity, off-target effects

## Abstract

The induction of robust CD8 T cell immunity after intramuscular (i.m.) mRNA vaccination has remained a challenge. Due to the limited presence of professional antigen-presenting cells (APCs) in muscle tissue, this route of administration tends to result in the transfection of muscle cells at the injection site with insufficient T cell activation capacity. The attraction of migratory APCs and related processes that lead to the acquisition of antigenic material from transfected non-APCs arises as a potential alternative to facilitate activation of CD8 T cells in the draining lymph nodes. This indirect pathway, known as antigen cross-presentation, has remained underappreciated for mRNA vaccines. This review provides a comprehensive analysis of this process. Due to the paucity of information available in this context, it also extrapolates from insights for antigen cross-presentation more generally and for traditional vaccines. Arguments are provided as to why this natural process in the context of pro-drugs, such as mRNA vaccines, may engender both specific and nonspecific responses and, in certain situations, evoke cross-tolerance rather than immunity. This widely unaccounted T cell activation process may, therefore, explain several key mysteries surrounding i.m. RNA vaccination, including its impact on heterologous infections. But it also raises numerous open questions that are clearly described.

## 1. Introduction

Although billions have undergone COVID-19 mRNA injection and numerous mRNA vaccine platforms are in development for various other applications, we still lack a basic understanding of their cellular uptake, endosomal escape, fate, and mechanisms of immune induction. Many studies have sought to uncover how adaptive immunity emerges, revealing numerous paradoxical findings. For example, mRNA vaccines have shown mysterious effects on heterologous infections [1,2,3,4,5]. Although this is ascribed to trained innate immunity, the underlying immune processes are unknown. When aiming to elucidate the key immunological players, Alameh et al. [6] discovered an underappreciated role of the adjuvanticity of the LNP component linked to its inherent inflammatory nature. Yet, further studies have shown that some versions of mRNA vaccines have proven protective even when non-inflammatory LNPs were used [7]. More fundamentally, questions have also emerged about the underpinnings of conventional immune processes and the key cells they rely on. In this regard, Ndeupen et al. [8] discovered that the mRNA-LNP platform can support the induction of protective immune responses without key innate immune cells and cytokines that traditionally play a central role in inducing antibody responses.

After a virus enters a host and infects cells, rapid and effective clearance of the infection is mediated by CD8+ cytotoxic T lymphocytes (CTLs). CTLs recognize infected or transformed cells by detecting major histocompatibility complex (MHC) class I molecules on their surface, which present antigenic peptides derived from viral proteins or mutated gene sequences. In turn, the CTL then kills the offending cells, thereby eliminating the source of viral replication or the abnormal/cancerous cells.

During the development and rollout of COVID-19 mRNA vaccines, B cell immunity has drawn the most attention. However, mRNA vaccine-induced humoral immunity against SARS-CoV-2 wanes [9]. On the other hand, relatively little else is known about how mRNA vaccines impact CD8+ T cell responses. Specifically, this has prompted the development of several entirely new platforms intended to contribute to more durable and effective CD8+ T cell immunity. In part, these new approaches were also motivated by particular obstacles identified with the COVID-19 mRNA injections. Recently, Fang et al. [10] observed that deficiencies in the encoded protein’s subcellular localization often impair immunity. Although their optimized platform enhanced antibody responses, cellular immunity showed no consistent pattern, with some T cell responses even reduced. Likewise, the novel mRNA vaccine platform by Aunins et al. [11], which specifically aims to enhance CD8+ T cell responses, reports deficiencies with the inherent adjuvants of the COVID-19 mRNA vaccines and how this affects cellular immunity. Nevertheless, whilst suggesting an optimized response, their new method raises questions as well.

Intravenous delivery of mRNA cancer vaccines elicits a stronger antigen-specific CD8+ T cell activation than local injection routes [12]. While cancer vaccines aim to induce a powerful systemic cytotoxic T cell response, such widespread immune activation is undesirable in prophylactic vaccines due to the risk of excessive inflammation. Therefore, intramuscular injection modalities face greater challenges in stimulating effective CTL responses without triggering significant inflammatory reactions.

Another dilemma with mRNA vaccines is that their modus operandi unfolds inside cells. The process by which encoded antigens reach MHC presentation is complex, especially when transfected cells are not professional antigen-presenting cells (APCs). Thus, if the APC is not itself synthesizing the antigen, as is often the case with mRNA vaccines, it must acquire the antigen exogenously in a process termed cross-presentation.

Generally, cross-presentation is carried out by professional APCs, especially dendritic cells (DCs), which can internalize antigens not generated within themselves but acquired from other cells. This process plays a crucial role in health and disease, significantly expanding the range of antigens that can be presented via the MHC I pathway.

Accordingly, naïve CD8 T cells can be primed by professional APCs, or by direct antigen presentation or (indirect) antigen cross-presentation [13]. In the case of direct antigen presentation, which applies to antigens expressed in professional APCs, such as from viral infections, these right away enter the classical MHC class-I pathway of antigen processing and presentation of antigenic peptides. During the development and rollout of the mRNA vaccines, it seems that the cellular immune responses via this canonical (direct) T cell activation and differentiation pathway were taken for granted.

Nonetheless, prior vaccines have heavily relied on the alternative antigen cross-presentation pathway whereby antigenic material introduced into the extracellular space or derived from principally any cell type is taken up by professional APCs and eventually fed into the MHC class-I pathway. Paradoxically, this indirect pathway has received little attention in the context of mRNA vaccines. As of 27 August 2025, a PubMed search revealed no studies on COVID-19 mRNA vaccines that examined antigen cross-presentation (summarized in Table 1). Although this term was briefly mentioned in some articles, any relevant experiments had been performed at least ten years prior.

To help fill this gap, this article provides the first comprehensive review of antigen cross-presentation in mRNA vaccines, focusing on intramuscular (i.m.) administration. Key aspects of this study are based on a review and analysis of the following:

- Available information for mRNA vaccine antigen cross-presentation in the literature.
- Insights derived about antigen cross-presentation in general, the uniqueness of mRNA vaccines as a pro-drug, what this might mean in this regard, and novel open questions.
- Extrapolation of potential mechanisms, pathways, and triggers.
- The limited direct evidence of mRNA vaccine cross-presentation that has been published, and a summary of indirect evidence.
- The good, bad, and ugly of antigen cross-presentation and unique implications for intramuscular mRNA vaccines.
- Technical challenges in harnessing antigen cross-presentation for mRNA vaccines and differences to protein and subunit vaccines.
- Potential overlaps or explanations to unanswered findings about these technologies, including immunity towards heterologous infections and off-target T cell responses.

## 2. Antigen Cross-Presentation—A Promising Modality for CD8+ T Cell Activation Following COVID-19 mRNA Vaccines, but with Unique Challenges

Inducing robust CD8+ T cell immune responses is an important goal pursued by both cancer immunotherapies and infectious disease vaccines. As noted, extracellular antigens require cross-presentation to be presented by MHC-I. While at least two pathways can achieve this, the primary route is the cytosolic pathway. In this case, the antigens acquired by APCs are delivered to the cytosol and subsequently transported into the ER, where peptides are further trimmed and loaded onto MHC-I [23].

Significant research has been devoted to the indirect T cell activation process for subunit, protein, and peptide vaccines. In those situations, antigen cross-presentation is necessary for the induction of CD8 T cell immunity because these vaccines deliver extracellular antigens that cannot directly enter the classical pathway of MHC class-I presentation. Unfortunately, mRNA vaccines, too, may release/secrete their antigenic material, especially during the transfection of non-APCs. Although this suggests indirect T cell activation, a validation of this possibility, as performed with more traditional vaccines, does not seem to have been conducted.

### 2.1. The Predicted Route for i.m. mRNA Administration: Direct CD8+ T Cell Activation

In the context of i.m. mRNA vaccines, focus has mostly been on the activation of CD8+ T cells via the generation of antigenic peptides generated directly in an APC-transfected cell. This process is thought to be facilitated by the local inflammation created by the platform, which drives recruitment of neutrophils and APCs to the site of delivery. The recruited immune cells can, in turn, acquire the mRNA-LNPs and then migrate to local lymph nodes.

Specifically, the EMA assessment report [24] emphasizes that “recruited APCs are capable of LNP uptake and protein expression and can subsequently migrate to the local draining lymph nodes [dLN] where T cell priming occurs.”

### 2.2. A Key Dilemma with i.m. Administration

Again, according to the EMA, a critical aspect of COVID-19 mRNA vaccines is their capacity for direct APC transfection. Unfortunately, the pathways for this were not demonstrated in [24]. It is possible that i.m. mRNA-LNP injection causes low-level transfection of APCs at the injection site, which then migrate to lymphoid organs to present the encoded antigen to B and T cells. However, it has been found that after i.m. mRNA-LNP administration, the predominant cells that become transfected are the local muscle fibers at the injection site, which then produce the encoded protein [25].

Since myocytes as non-professional APCs express MHC class I at low levels and do not constitutively express class II or co-stimulatory molecules [26,27], they are unlikely to support a strong induction of cellular immunity.

### 2.3. Direct Transfection of Dendritic Cells by COVID-19 mRNA Vaccines, as Commonly Assumed

**Figure 1:**
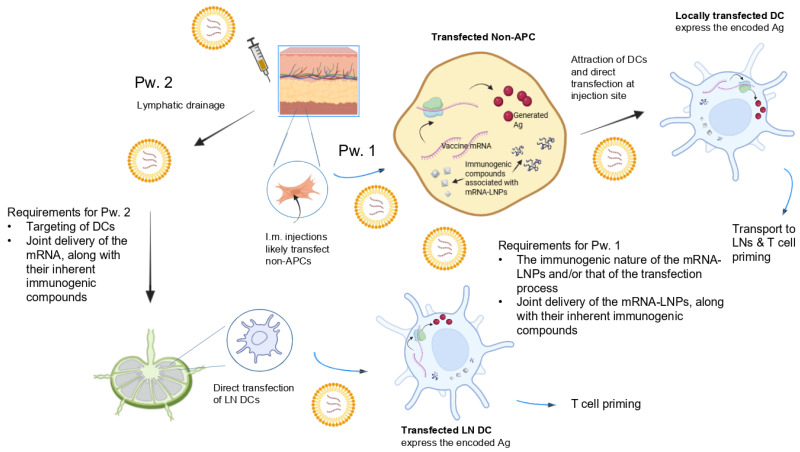
Commonly understood pathways of how i.m. RNA vaccines activate cellular immunity. At the heart of this approach is the transfection of professional immune cells, even though injection-site-transfected cells are primarily somatic. Consequently, the encoded antigen is expressed directly in professional APCs, which, in the lymph nodes, leads to T cell priming. Created in BioRender. Mueller, S. (2025) https://BioRender.com/v52b520 (accessed on 22 September 2025).

Given that i.m. injection mostly results in the transfection of non-APCs, according to the above EMA expectation, mRNA-LNPs must both attract and directly transfect APCs (mostly DCs). It has been postulated [28] that this is possible via two routes (summarized in Figure 1). First, transfection of injection site cells may facilitate the rapid recruitment of immune cells to the injection site. Internalization of mRNA-LNPs at the injection site by APCs would trigger protein expression and APC transport of the expressed antigen to draining LNs (dLNs). Second, mRNA-LNPs may drain to the dLNs and directly transfect the APCs in those compartments.

Indeed, in rodents and non-human primates, mRNA and expressed protein have been found in both tissue-resident/infiltrating immune cells at the injection site and in draining lymph nodes [28]. This indicates that i.m. mRNA-LNP injections can travel directly via the lymphatic system to LNs, without necessarily transfecting cells at the injection site first.

However, the extent to which this is possible remains questionable. Actually, in recent years, the observation that i.m.-administered mRNA vaccines poorly transfect immune cells has triggered considerable research on new carriers for a more effective direct transfection of DCs, e.g., by targeting their unique surface markers [25].

SARS-CoV-2 spike mRNA vaccines to control the pandemic are generally formulated with lipid nanoparticles (LNPs), which are composed of several lipids with specific ratios; however, they generally lack selective delivery [29]. With safety being a critical aspect, it is generally accepted that transfection away from the injection site would be undesirable [30].

To date, the selective targeting of mRNA into immune cells has remained an unresolved issue related to mRNA vaccine immunity. Ongoing research efforts strive to enhance the efficacy of the immune response. Simultaneously, it is necessary to reduce unwanted off-target biodistribution and its implied adverse sequelae [2,31,32,33,34].

### 2.4. The Core Challenge of CD8+ T Cell Responses Following i.m. mRNA-LNP Immunization May Be Bypassed by Antigen Cross-Presentation

#### 2.4.1. Foundational Insights About Self-Amplifying mRNA (SAM) Vaccines

Ten years ago, Lazzaro et al. [21] conducted a detailed study to analyze the priming of CD8 T cells by self-amplifying mRNA (SAM) vaccines. They found strong evidence for antigen cross-priming as the prevalent mechanism. In their experiments, they validated this as follows.

- Muscle cells served as the main site of antigen expression.
- These cells could not directly prime CD8 T cells.
- CD8 T cell priming upon SAM vaccination was restricted to bone marrow-derived (BM) APCs, such as DCs, rather than direct transfection of APCs themselves.

Thus, the key mechanism was described as antigen transfer from transfected myocytes to BM-derived APCs to enable MHC class-I-restricted presentation to CD8 T cells via antigen cross-presentation. Lazzaro et al. also revealed profound details, suggesting the following mechanisms:

- Migration of APCs toward the antigen-expressing cells and direct intercellular transfer.
- Apoptosis of transfected cells triggered by the high-level replication of the SAM mRNA in the transfected cells.
- Phagocytosis of apoptotic bodies released when the transfected cells undergo cell death.

Lazzaro et al. also suggested potential triggers that activate the migration of APCs to the injection site. They envision that one of the following induces soluble factors that may promote APC migration: (a) RNA transfection itself, (b) replication of the transfected RNA or antigen, or (c) antigen expression by the transfected cells. As for antigen uptake, interestingly, they found that these BM-DCs acquired both the antigen and dsRNAs from the transfected cells.

Granted, SAM vectors encode replicase genes that drive high-level, self-sustained replication of the mRNA, producing double-stranded RNA (dsRNA) intermediates. However, some concepts proposed by Lazzaro et al., when compared to non-SAM platforms, strongly indicate similar mechanisms for CD8+ T cell activation and CTL induction in COVID-19 mRNA vaccines (Table 2).

#### 2.4.2. Both Cross-Presentation and Direct APC Transfection Are Possible for DNA Vaccines

Already 25 years ago, it was known that both cross-priming and direct transfection of APCs play a role in DNA vaccines. Regarding the former, there is concrete evidence that these vaccines exploit the ability of immature DCs to endocytose soluble proteins and debris from apoptotic cells and then present these antigens in the context of MHC class-I molecules to facilitate the cross-priming of CTLs [44]. Many of the key observations in this study parallel those identified by Lazzaro et al. [21] for the SAM mRNA vaccines.

- In the case of i.m.-injected DNA vaccines, myocytes were found to be the main cell type transfected. Despite the predominance of these non-immune cells, the injections yielded potent cell-mediated immunity.
- Apparently, in this context, the induction of CTL responses was facilitated by antigen transfer from myocytes to professional APCs. In fact, the production of antigens by muscle cells alone was sufficient to induce CTL responses. Likewise, DNA vaccines that employed a muscle-specific promoter were able to induce CTLs in mice. Notably, in this case, CTLs were primed by antigens that were expressed only in non-APCs (i.e., muscle cells).
- The observation that antigens synthesized in non-APCs were transferred to professional APCs for presentation in the context of MHC I means that direct transfection of APCs by DNA vaccines is not required for immune activation.

### 2.5. Uncertainty of Whether Some mRNA Vaccine Studies Implicitly Involve Antigen Cross-Presentation

Experiments have shown that LNP mRNA vaccines lack cell specificity, particularly for DCs [45]. This highlights the need for an LN-targeting LNP mRNA vaccine with DC tropism. Unfortunately, as noted earlier, directly transfecting DCs has proven to be much more challenging than initially anticipated. Still, it is not apparent that this has led to a re-evaluation of the expected pathways first proposed by the EMA (Figure 1).

Unexpectedly, research seems to have confirmed the notion that, in this case, CD8+ T cell activation happens mainly via direct transfection of DCs or other APCs. Indeed, several studies have reported that DCs and other APCs not only internalize mRNA-LNPs but also translate the mRNAs. However, these studies generally do not clarify whether the vaccine-encoded antigen peptides are derived from local translation or from antigens produced in transfected non-APCs.

Nonetheless, colocalization of the expressed protein and immune cells in dLNs is no proof of direct transfection of APCs. Could the prevailing view, seemingly accepted by the EMA, have shaped expectations so that the alternative pathway remains underappreciated?

In retrospect, some of the existing research could plausibly support both of these pathways. For example, Kim et al. [35] reported that mRNA-LNPs recruit migratory DCs, which express high levels of IFN-stimulated genes both at the injection site and in the draining lymph nodes. While this reflects an innate immune activation signature in those cells, it does not demonstrate that the vaccine mRNA is actually translated into protein antigens within the lymph nodes.

Actually, it seems that the direct pathway is often implicitly assumed. For example, in 2017, Liang et al. [34] claimed to have identified direct mRNA uptake and translation in APCs based on experiments in rhesus macaques involving intramuscular or intradermal injections of fluorescently labeled LNPs containing mRNA encoding a reporter protein. While the localization of the signals could support direct translation in monocytes and DCs, the study lacks exclusionary experiments that would definitively rule out antigen transfer from neighboring cells. Similarly, very recent analogous findings [46] do not explicitly rule out antigen cross-presentation either.

Therefore, the contribution of the direct vs. the indirect pathway in CD8+ T cell activation remains to be further elucidated. To distinguish direct translation in DCs from antigen transfer from somatic cells at the injection site, comparative assays (e.g., blocking somatic cell expression, using translation inhibitors in DCs, or other cell-specific knockout models) would be needed to exclude cross-presentation.

### 2.6. Antigen Cross-Presentation in mRNA Vaccines—Potential Mechanisms

Ironically, one of the main challenges with i.m. mRNA-LNP administration is that they do transfect cells at the injection site. As noted, these predominantly non-immune cells, in this compartment, are not directly able to facilitate potent cellular immune activation.

With i.m. mRNA vaccines, it is, therefore, necessary that the antigenic compounds from principally transfected muscle cells can be presented by professional APCs in the LNs, along with appropriate pro-inflammatory signals. Due to the scarcity of studies on antigen cross-presentation in licensed mRNA vaccines (Table 1), few details about this process are reported in the literature. However, processes and mechanisms of antigen cross-presentation in general are well documented and involve phagocytosis, pinocytosis, or receptor-mediated endocytosis [47,48]. Extending these to the context of mRNA vaccines, it is reasonable to assume that the processes indicated below facilitate antigen transfer from non-immune to immune cells and, in turn, trigger antigen cross-presentation.

#### 2.6.1. Antigenic Transfer from Transfected Somatic Cells to Professional APCs—Potential Mechanisms

**Figure 2:**
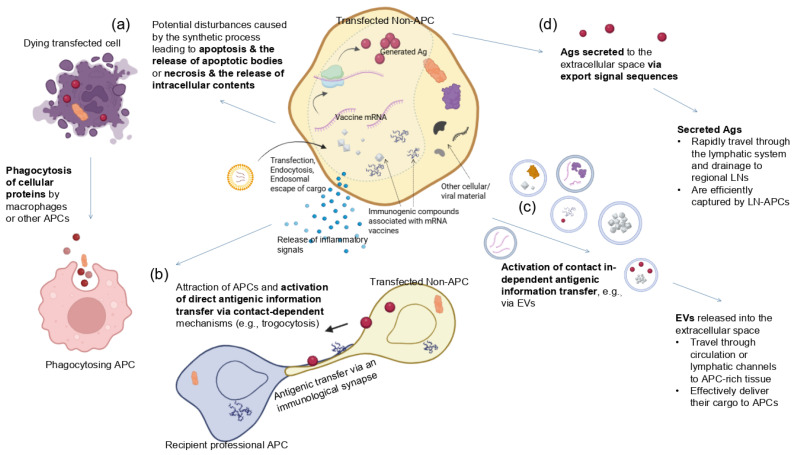
mRNA vaccines may naturally engage antigen cross-presentation processes. The figure depicts various mechanisms postulated to facilitate the transfer of antigenic material from transfected non-APCs to professional immune cells. In (**a**), antigenic transfer from the principally transfected non-immune cell is initiated by various “eat-me” signals triggered by the mRNA vaccine ingredients or the transfection process itself; specifically, potential disturbances initiated by the synthetic materials and resulting processes may trigger cell death, engendering the acquisition of various cellular antigenic material by the recipient APC. In (**b**), signals evoked by the transfection process, e.g., via the intrinsic inflammatory nature of the vaccine components, may attract professional APCs and activate antigenic information transfer via contact-dependent mechanisms, such as via trogocytosis or gap junctions. In (**c**), antigenic material can also move from transfected non-APCs to professional APCs through contact-independent pathways, via exosomes and other extracellular vesicles (EVs). These vesicles can effectively ferry diverse antigenic components to immune-rich tissues and organs. Finally, mechanism (**d**) exploits the fact that some vaccine antigens are equipped with export signal sequences to facilitate their presentation on the cellular surface; in turn, some of these antigens may be secreted into the extracellular space, making them amenable to rapid transport to draining lymph nodes via the lymphatic system. Created in BioRender. Mueller, S. (2025) https://BioRender.com/v52b520 (accessed on 22 September 2025).

Several mechanisms can be envisioned in which antigenic material from transfected non-immune cells is transferred to cross-presenting DCs or other APCs (summarized in Figure 2).

(a) Processes related to transfection, endocytosis, endosomal escape, translation of the synthetic mRNA, incomplete mRNA degradation, etc., may trigger **apoptosis** of the transfected cells, leading to the formation and release of apoptotic bodies. Indeed, apoptotic bodies, as membrane-bound vesicles containing cellular contents, facilitate phagocytosis by professional APCs. Additionally, it seems plausible that disturbances following transfection with the mRNA platform [38,49] lead to necrosis of the transfected cell. The release of intracellular contents into the extracellular space would also trigger their uptake by immune cells via phagocytosis. Besides the encoded protein, the cellular debris acquired by APCs may also include genetic material, cytoplasmic proteins, and other intracellular molecules.
(b) The inherent adjuvanticity of mRNA-LNP components is known to lead to the production of chemokines, proinflammatory cytokines, and type-I interferons (IFN-I) and the attraction of immune cells [6,31,35,43,50]. First, the inflammatory milieu may activate **contact-dependent antigenic information transfer**, whereby cells in close contact can directly “nibble” material from living cells (“trogocytosis”) or related contact-dependent pathways that are activated under stress or infection [51]. Trogocytosis involves the acquisition of membrane/membrane proteins or other material from another cell. It has emerged as a form of cell–cell communication and cell signaling in the immune system [26] and was first suggested as a potential mechanism for antigen cross-presentation in 2005 [48]. Although it is widely involved in antigen presentation, information transfer, and immune cell regulation, not much is known about this modality [52]. Gap junctions, another contact-dependent antigen transfer, also do not require the donor cell to be an antigen-presenting cell and can involve infected or apoptotic cells [53]. Besides the vaccine antigen, the transfer may also include nucleic acid, peptide major histocompatibility complex (p-MHC), co-stimulatory molecules, tumor antigens, and the antigens of pathogens [51,52]. Antigen transfer often involves DCs as receptor cells to elicit potent immune responses. However, depending on the form of the transferred antigen and the maturation state of the receptor DCs, this can also promote central tolerance.

(c) Antigenic information transfer may, alternatively, be realized via **contact-independent** extracellular vesicles (EVs) [51]. EVs are small spherical lipid bilayer particles released into the extracellular environment by almost all types of cells. These biological carriers, including exosomes, are known for their ability to effectively transmit various cargos such as lipids, proteins, or nucleic acids between cells, tissues, and even across species and kingdoms [54,55]. In the case of tumor cells or virus-infected cells, it is well established that antigen transfer to DCs via extracellular vesicles plays a crucial role in initiating and sustaining specific immune responses [56]. Antigen cross-presentation via synthetic exosomes has long been explored as a synthetic vector for vaccine development [57]. Interestingly, DCs can selectively engulf EVs incorporating antigenic compounds. On the other hand, EVs with insufficient co-stimulatory signals and/or adjuvant-like components may induce immune tolerance when internalized by immature DCs [27].
(d) Antigens can be released into the extracellular space through alternative mechanisms, such as cell surface translocation or export sequences that prevent them from remaining trapped inside the cell. The modulation of this process has very recently been suggested via novel mRNA vaccines that encode antigens with optimized signaling information to facilitate their extracellular translocation [10].

Overall, the phagocytosis mechanism is the most extensively studied for antigen cross-presentation. In fact, the release and acquisition of cellular proteins secreted or released from dying/impaired cells was one of the first major antigen cross-presenting mechanisms identified [48]. Phagocytes quickly engulf dying cells, internalizing debris into phagosomes that play a key role in cross-presentation. And, intriguingly, dead cells were long thought to function as an adjuvant because they release “danger signals” [48].

In all (Figure 2), several mechanisms can be envisioned of antigenic transfer from transfected somatic cells to professional APCs, such as DCs. Some of these exploit the well-known ability of tissue DCs to transport antigens to secondary lymphoid organs, thereby facilitating CTL-cross-priming [58]. Additionally, secreted antigens (Figure 2d), directly traveling through the lymph, likely lead to antigen cross-presentation in a relatively short time frame.

#### 2.6.2. Pathways for MHC Class-I Antigen Presentation

Generally, the exogenous antigenic material acquired by professional APCs from transfected somatic cells can be processed in different ways [47]. Once internalized by APCs, the antigen can be processed through two main mechanisms [48]. In the vacuolar pathway, an exogenous antigen is endocytosed and degraded into peptides via lysosomal proteases, generating epitopes that are directly loaded onto MHC-I on the vesicle membrane.

In the endosome-to-cytosol pathway, antigens are transferred into the cytosol, where they undergo proteasomal degradation. Afterward, antigen-derived peptides are transported back into the endosomes or into the ER via the transporter associated with antigen processing (TAP) and loaded onto MHC I [53,59].

**Figure 3:**
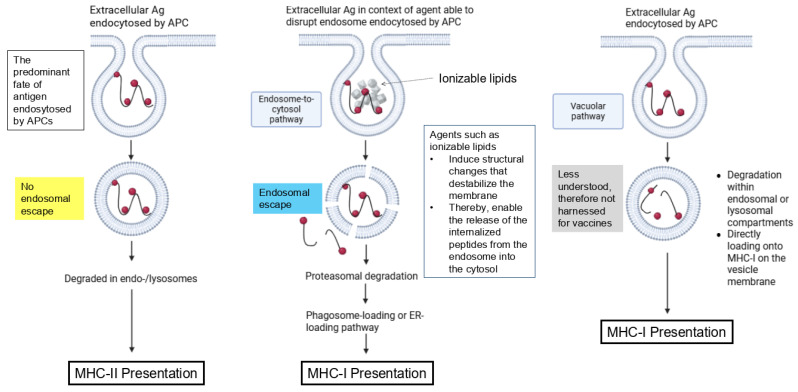
Summary of the primary pathways for T cell activation by extracellular antigens (Ag): the MHC-II pathway leads to activation of CD4+ T cells; accordingly, various vaccine platforms have targeted key steps to promote MHC-I presentation and thereby elicit CD8+ T cell activation. Figure adapted from [59]; used with permission (License Number 6111520931191). Created in BioRender. Mueller, S. (2025) https://BioRender.com/v52b520 (accessed on 22 September 2025).

It does not seem that details about these processes have been studied for mRNA vaccines. Alternative vaccine platforms have aimed to specifically harness antigen cross-presentation, e.g., via molecular engineering and nanotechnology [59]. The following have proven critical, showing that mRNA vaccines may be more prone to this process (Figure 3):

- The predominant fate of antigens endocytosed by APCs is degradation in the endo-/lysosome and presentation on MHC-II for priming of CD4+ T cells. Therefore, specific vaccine platforms have pursued steps to bypass endosomal trafficking of exogenous antigens and to promote delivery of antigens to the cytosol of APCs.
- To potentiate an antigen-specific CD8+ T cell response, immune-stimulating adjuvants are required.
- Considerable effort has been dedicated to designing mRNA vaccine platforms that enhance endosomal escape in transfected cells, for example, through their intrinsic adjuvants and ionizable lipids. Analogously, these compounds, when acquired by APCs (Figure 2), can also destroy endosomal membranes in the cross-presenting cell. As a result, mRNA vaccines may inherently promote certain antigen cross-presentation mechanisms more effectively than alternative vaccines.
- The vacuolar pathway is less understood and, for more traditional vaccines, is not targeted, as this would also require increased antigen uptake and retention. On the other hand, this pathway may be more accessible to mRNA vaccines because of their prolonged antigen persistence compared to other platforms, as well as their inherent adjuvant properties.

## 3. Antigen Cross-Presentation of COVID-19 mRNA Vaccines

### 3.1. Antigen Cross-Presentation Not Widely Expected

As detailed above, it has been widely assumed that the mRNA vaccines, despite being taken up by immune and non-immune cells, activate immunity upon direct transfection of professional APCs and presentation of the vaccine antigen on their surface [60]. For example, Chaudhary et al. [60] seem to take it for granted that the vaccine antigen is produced intracellularly in APCs, processed into smaller fragments, and directly presented to CTLs by MHC class I molecules.

They mention the potential of vaccine antigens to be secreted and acquired again by immune cells. As indicated, extracellular antigens that are endocytosed by APCs will be predominantly degraded in endo-/lysosomes and presented on MHC-II molecules [59]. Therefore, with mRNA vaccines, it was apparently assumed that this canonical pathway of exogenous antigens would be the predominant one. Nonetheless, exogenous antigens, once internalized by APCs and processed and loaded onto MHC class-II molecules, activate CD4+ T helper cells [61].

On the other hand, the classical direct pathway for CD8 T cell activation relies on endogenous antigens, where peptides derived from intracellularly synthesized proteins are presented on MHC class I molecules. As detailed before, for mRNA vaccines, this was believed to be realized by direct transfection of APCs. Yet, when mRNA vaccines trigger the secretion and release of antigens, by virtue of their nature as exogenous antigens, they cannot directly enter the classical pathway of MHC class-I presentation. The possibility of cross-presenting exogenous antigens through MHC class I molecules as a means to activate naïve CD8 T cells may have been deemed unlikely. This indirect pathway was briefly suggested, e.g., in [62,63], but, seemingly, mainly as a theoretical possibility without any specific evidence for the mRNA injections.

### 3.2. Direct Evidence

A landmark study published in 2024 [35] successfully characterized the early immunogenic signature following injection and connected the innate and adaptive immune responses. Kim et al. analyzed the responses of specific fibroblast subtypes at the injection site by enriching for mRNA vaccine transcripts in these cells. In turn, they conducted pathway enrichment analysis on the differentially expressed genes (DEGs) of the main fibroblast populations involved. This revealed that one of the main pathways identified in a specific fibroblast population (Fib_Cxcl5), depicted in Figure 3j in [35], is

“antigen processing and presentation of exogenous peptide antigen via MHC class I” and “antigen processing and presentation of exogenous peptide antigen via MHC class I, TAP-dependent.”

The authors did not comment on this. It may have been surprising that, rather than the expected [63] targeting by class-I MHC proteins to CTLs of intracellular proteins, this involves class-I MHC-presented peptides generated from exogenous proteins. Nonetheless, the following hold:

- These experiments provide strong evidence of antigen cross-presentation after mRNA vaccination. They also show involvement of the two main pathways of this process.
- The detailed kinetics suggest that antigen cross-presentation induced by mRNA vaccines can be triggered within hours of i.m. administration.
- This indirect process of antigen processing and presentation may engage an unexpected non-immune cell type (fibroblasts).

#### 3.2.1. Antigen Cross-Presentation via Injection Site Fibroblasts

The pathway enrichment analysis by Kim et al. was specific to fibroblasts. Usually, it is understood that cross-presentation is facilitated by DCs but also by macrophages and B cells [64]. Regardless, the potential of fibroblasts for antigen cross-presentation has actually been noted before.

#### In the Lab, Fibroblasts Can Be Reprogrammed into DCs Through the Ectopic Expression of Specific Transcription Factors

Interestingly, in certain laboratory studies, fibroblasts have been successfully converted into DCs, acquiring their characteristic ability for cross-presentation and immune activation. Notably, Rosa et al. [65] demonstrated that mouse and human fibroblasts can be reprogrammed into functional DCs capable of engulfing, processing, and presenting antigens to T cells and capable of secreting inflammatory cytokines. Interestingly, the murine-induced DCs also acquired the capacity for potent capture of exogenous antigens and cross-presentation to CD8+ T cells in the context of MHC-I molecules.

Their work involved “reprogramming” somatic cells by transducing them with key transcription factors to alter their transcriptional and epigenetic states. Even though fibroblasts are thought not to naturally transform into DCs, it is tempting to speculate that unaccounted “rewiring” could also be evoked by the mRNA injections in vivo, via their capacity to induce strong and unique transcriptional responses [35] that are only beginning to be elucidated.

#### Cross-Presentation in Fibroblasts in Mice and Humans

In mice, it has been shown that several non-professional APCs, including cancer-associated fibroblasts (CAFs), possess an antigen cross-presentation capacity [66]. Harryvan et al. [64] were able to extend these findings to humans. They demonstrated that human CAFs in the context of colorectal cancer have an enhanced potential to cross-present neoantigen-derived synthetic long peptides (SLPs) compared to normal colonic fibroblasts. Moreover, cognate interaction between CD8+ T cells and cross-presenting CAFs suppressed T cell function, as indicated by decreased cytotoxicity, reduced activation (CD137), and increased exhaustion (TIM3, LAG3, and CD39) marker expression. These suggest that, at least in the case of some colorectal cancer-derived fibroblasts, these cells are capable of antigen cross-presentation and suppression of tumor-specific T cell function in an antigen-dependent manner both in mice and humans.

#### 3.2.2. The Study by Li et al. [43]

Interestingly, Li and colleagues [43] also emphasized that cross-presentation is critical for BNT162b2-induced T cell responses. Unfortunately, they did not provide any further details in this regard.

### 3.3. Indirect Evidence

Synthetic exosomes, capable of carrying diverse antigens, are explored as alternative vaccine carriers [67]. Antigens in exosomes are efficiently cross-presented by DCs [68,69]. Therefore, although there is no conclusive proof of antigen cross-presentation, endogenous exosomes carrying antigens can serve as an indicator of this process. Several studies about COVID-19 mRNA vaccines have demonstrated the involvement of exosomes or other EVs carrying vaccine antigens, vaccine mRNAs, or other vaccine compounds (reviewed in [7]). Examples include the following:

- One of the first studies that revealed the pivotal role of exosomes with mRNA vaccine immunity was performed by Bansal et al. [37]. Their work initially triggered criticism [70] regarding the prolonged persistence of the vaccine antigen, which, however, is no longer controversial [71]. Aside from the kinetic considerations, they not only confirmed the presence of the Pfizer vaccine antigen in exosomes but also envisioned how the induction of the immune responses required the antigen to be presented in exosomes. Implicitly, their proposed mechanism utilizes cross-presentation via exosome-facilitated antigen transfer from transfected cells to professional APCs. Congruent with the above, they reasoned that the induction of circulating exosomes with the SARS-CoV-2 spike protein antigen is necessary for effective immunization following mRNA-based vaccination.
- Kämmerer et al. [38] identified that the vaccine antigen was physically shown to be packaged into exosomes secreted from host cells exposed to the vaccine in culture.
- Hanna et al. [39] studied whether the COVID-19 vaccine mRNA can be detected in the breast milk (BM) of lactating women who had received the vaccination. The study provided evidence for the presence of vaccine mRNA in EVs isolated from milk supernatants. They highlighted the potential ability of tissue EVs to package the vaccine mRNA, or fragments thereof, and transport it to distant cells.

Prior studies that found EVs carrying vaccine mRNAs were mainly concerned about their potential to produce vaccine antigens in distant tissues. Still, their potential involvement in antigen cross-presentation does not seem to have been studied. As detailed below, the modified mRNA’s inherent adjuvant effect on immune cells may inadvertently enhance CD8+ T cell responses to untargeted (self)proteins.

## 4. The Role of Cellular Localization, Co-Stimulation, and Tolerance

Given that the current paradigm widely underappreciates the potential involvement of antigen cross-presentation, is it possible that this has resulted in unaccounted consequences? Different pathways may activate variably across contexts, uniquely shaping immune responses. Below, specific considerations will be highlighted, informed by this notion, that indicate aspects that, to date, do not seem to be widely known.

### 4.1. Apparently Conflicting Results About mRNA Vaccine Cellular Immunity

During the development and rollout of the mRNA injections, the involvement of vaccine-induced T cell immunity did not receive the same level of attention as B cell immunity. Although T cell immunity is widely regarded as crucial, even in the year 2022, there was no clear understanding of which T cell subpopulations protect against COVID-19 [72]. Meanwhile, published reports on mRNA vaccine-fostered T cell immunity have often resulted in conflicting or inconclusive results. For example, Pfizer’s BNT162b2 study for V8 and V9 in mice showed CD8+ and CD4+ T cell activation in splenocytes upon S protein peptide stimulation, measured by IFN-γ ELISpot [73]. However, compared to the control animals, they did not observe a significant cytokine increase in the repeat dose study in rats. One animal study reported high variability and cytokine increases in both control and treated groups. Similarly, their in vitro study using human peripheral blood mononuclear cells (PBMCs) gave inconclusive results.

Unlike Pfizer, Moderna’s COVID-19 mRNA vaccine did not induce CD8+ T cells against spike peptides, although both triggered CD4+ T cell responses [63]. For Moderna [74], CD8 T-cell responses were observed only at low levels after the second vaccination with the higher 100 μg dose.

Satija et al. emphasized the crucial role of T cell immunity in early mRNA vaccine protection against COVID-19, identifying distinct CD8+ T cell subsets emerging 28 days after vaccination that predicted clinical outcomes [75]. Likewise, a study conducted in Jordan [76] that contrasted T cell-mediated immunity and side effects of mRNA vaccines with conventional COVID-19 vaccines reported that the Pfizer-BioNTech vaccine induced higher helper CD4+ T cell responses compared to non-Pfizer-BioNTech vaccines. They argued that the CD3+/CD8+ (T cytotoxic) level was notably elevated in non-Pfizer-BioNTech recipients.

A different picture was described for immune-compromised individuals, where mRNA COVID-19 vaccines resulted in much higher levels of IgG4 antibodies and impaired activation of CD4+ and CD8+ T cells [77]. Similarly, Sureshchandra and colleagues [78] identified that CD8+ T cell responses following SARS-CoV-2 mRNA vaccines were relatively weak and variable. Interestingly, although clonally expanded CD8+ T cells were observed, the frequency of S-specific CD8+ T cells was small. Other sobering findings related to mRNA vaccine cellular immunity were recently published by Gimenez et al. [79]. They identified a highly destructive cascade of events triggered by the vaccine-induced circulating SARS-CoV-2 mRNA vaccine antigen in analogy to what is known during natural infection. The implication is monocytic release of reactive oxygen species, T cell death, and impaired cellular immune response to SARS-CoV-2 mRNA vaccines.

### 4.2. The Cellular Localization of the Vaccine Antigens and the Immune Activation Pathway Triggered

The reasons for the above disparate results are likely multi-factorial. The following sections argue that antigen cross-presentation may explain some of these inconsistencies and other unexpected observations.

#### 4.2.1. DNA Vaccines Require Antigen Cross-Presentation for CD8+ T Cell Priming

For genetic vaccines, the role of antigen cellular localization in CD8+ T cell activation was first demonstrated with i.m. DNA vaccines. As mentioned, it had previously been established that these modalities activate immune responses via both direct and indirect pathways [26].

Surprisingly, when the role of the cellular location of the expressed antigen was examined [26], it was found that immune responses were greatly improved for secreted antigens compared to the cytoplasmic or membrane-bound forms. The authors suggest that this indicates that the canonical class-I pathway, despite its ready access to cytoplasmic antigens, is less effective for antigen presentation. On the other hand, antigens that were secreted, e.g., by transfected non-APCs, engage the indirect CD8 T cell activation pathway. The improved immune response in [26] indicates that effective priming of CTL responses after i.m. DNA immunization relies on antigen cross-presentation rather than direct presentation.

Interestingly, these experiments also revealed that after i.m. DNA vaccination, the vaccine DNA is primarily taken up and expressed by myocytes near the injection site. In this sense, the myocytes likely merely act as a source of antigen or immunogenic material to initiate antigen transfer to cross-presenting APCs, which facilitate priming in the lymph nodes. This could explain why in [26] the immune response was diminished when the antigen was cytoplasmic.

#### 4.2.2. Cellular Localization of mRNA Vaccine Antigens Not Guaranteed

Experiments with DNA vaccines demonstrate that the intracellular localization of vaccine products—particularly whether they are secreted or retained in the cytosol—significantly influences immune recognition and activation. Unfortunately, the cellular localization of mRNA vaccines remains insufficiently understood.

Pfizer and Moderna modified the mRNA sequences to anchor the vaccine antigen to the APC membrane, aiming for a strong, lasting immune response. However, this presumes that the injections have succeeded in directly transfecting APCs. A potential anchoring to the cell membrane also raises questions when the transfected cells are non-immune cells, particularly since these do not have the full machinery for cellular immune induction.

Unfortunately, the cellular fate of the COVID-19 mRNA vaccine antigen was poorly understood before its deployment. For example, Pfizer [73] relied on drastically different settings with many details further blacked out. Their studies employed alternative test materials, such as a commercial transfection method and a modified pcDNA3.1 construct encoding P2S, instead of the actual vaccine. Moreover, it was further purified to enable demonstration of the expected prefusion conformation [7]. Key immune activation pathways of the actual vaccine formulations were not clearly demonstrated.

A February 2025 *Nature Communications* study introduced an mRNA-encoded PET reporter gene to visualize the distribution and persistence of antigen expression from mRNA vaccines [80]. The authors contend that this new construct was essential because earlier methods for tracking the cellular localization and fate of vaccine antigens suffered from considerable limitations. Whereas these either tracked the LNP, the nucleic acids, or the mRNA transcript, Blizard et al. correctly noted that “none of these approaches enable in vivo observation of the downstream mRNA translation and corresponding protein expression, which produces the key bioactive species.” As part of their work, they analyzed the cellular localization of some recombinant spike variants via surface staining of mammalian cells using respective antibodies against these proteins. However, for this part, they, too, relied on specific transfection of the spike proteins rather than the actual mRNA-LNP vaccine formulation. The reliance on explicit transfection rather than trafficking and uptake via the vaccine LNPs may indicate some unaccounted for contribution of the latter. Curiously, Blizard et al. [80] also identified variable surface trafficking patterns afforded by the different spike protein variants. This aligns with the differences in post-translational folding and processing seen in the antigens, which lack key features found in their viral counterparts [7].

From these studies, the fate of the vaccine antigens of the actual formulations remains undefined. Instead, the produced antigens are expected to manifest a variety of surface trafficking potentials depending on the unique cellular milieu encountered.

### 4.3. Potential Issues of mRNA Vaccine Antigens That Persist in the Cytosol

Somewhat unexpectedly, Kämmerer et al. found that the vast majority of mRNA vaccine antigens stayed inside the cell [38]. Only a small proportion was excreted, and, notably, mostly via EVs. This observation raises the following concern:

- The study demonstrated successful transfection in HEK293 cells, which are somatic cells. The study findings are congruent with the notion discussed before that non-professional APCs cannot activate T cells. Indeed, in [38], the majority of the encoded proteins remained inside the cells, impeding immune activation.
- Apparently, in this context, the encoded proteins are not recognized as antigens by the immune system. It remains unclear whether they are ignored by the immune system or trigger immune tolerance.
- Nonetheless, via their inherently inflammatory nature, the transfection process itself could still evoke antigen cross-presentation. This is evidenced in [38] by the engagement of EVs that secrete the antigenic material. It is reasonable to assume that this can be internalized by professional APCs for antigen cross-presentation.

The above raises the possibility that the encoded antigen may be retained within transfected non-immune cells. Whether and when this could, thereby, result in tolerance induction has not been defined. On the other hand, antigen cross-presentation could, potentially, rescue immune activation. However, without sufficient inflammatory and other signals, such a situation could also engender tolerance instead. The following section provides a more detailed discussion of this.

## 5. Cross-Immunity Versus Cross-Tolerance

One of the benefits of antigen cross-presentation is that it broadens the scope of antigens that can be targeted by CD8+ T cells. It enables the activation of cytotoxic T cells that would not normally recognize extracellular or non-immune cell-derived antigens. Nonetheless, even if this process leads to the activation of such cells by their cognate antigen and their proliferation, the effect is not automatically a protective immune response against these antigens.

In fact, cross-presenting APCs tend to induce or expand regulatory T cells (Tregs) rather than effector T cells when the APCs cross-present self-antigens or tissue-derived antigens without strong inflammatory or danger signals [81]. For a prophylactic vaccine, the effect would be the opposite of what is wanted.

### 5.1. Cross-Tolerance

The outcome of cross-presentation can be either tolerance or immunity. Which of these outcomes occurs is largely dictated by whether antigens are acquired by themselves alone, leading to tolerance (`cross-tolerance’), or with additional co-stimulatory and survival signals, which, via `cross-priming,’ lead to immunity [48].

For example, naive CD8 T cells that are stimulated by peptide–MHC complexes (immune activation signal 1) without co-stimulation (signal 2) may elicit tolerance instead of immune activation [23]. This indeed reflects the normal fate of T cells. Their tendency to die or remain unresponsive to self-antigens without co-stimulatory signals helps prevent excessive immune reactions and autoimmunity.

On the other hand, the full activation of APCs depends on the direct recognition of pathogen-associated molecular patterns (PAMPs) such as TLRs, RIG-I, and MDA5 [6], which is required to effectively support the survival and differentiation of naive CD8+ T cells into CTLs [82]. Indirectly activated APCs through inflammation can stimulate antigen-specific naive CD8+ T cell proliferation but cannot sustain their survival or drive cytotoxic T-lymphocyte differentiation. Importantly, this means that inflammation cannot substitute for direct recognition of PAMPs in CD8+ T cell priming.

Distinguishing between immunogenic and tolerogenic responses is a complex process. Factors that determine the direction of the immune response include ligand expression by the APC, the type and level of “danger signals” received, the involvement of CD4+ T cell help, and others. Additionally, even though higher levels of some cross-presented immunogenic antigens may induce tolerance, a lower level is likely ignored by naive T cells [83].

### 5.2. mRNA Vaccine-Induced Cross-Tolerance

Ordinarily, mRNA vaccines used as prophylactic measures are expected to engender immunity. Unfortunately, numerous facets, especially in the context of transfected non-immune cells, remain insufficiently known. Somatic cells do not express the co-stimulatory molecules or inflammatory signals required for complete CTL activation, often leading to tolerance or anergy instead of immune activation. Even if professional APCs acquire antigenic compounds from transfected non-APCs, this process still does not guarantee the acquisition of the signals required for cross-priming, making cross-tolerance a real possibility. mRNA vaccine-induced cross-tolerance may contribute to the observed decline in immune protection associated with this platform. Unfortunately, there is currently no indication that these potential connections have been examined.

Whereas in some contexts, cross-tolerance can be beneficial, it must be clearly regulated. Yet, with mRNA vaccines, these processes do not seem to have been sufficiently anticipated.

Cross-tolerance arises only when the cross-presented antigen dose and/or CTL affinity reach a critical threshold. If not, autoreactive CTLs may escape cross-tolerance and lead to disease when cross-primed [58]. Thereby, cross-priming has been implicated in type I diabetes, multiple sclerosis, and tumors. Determining whether these developments are associated with certain mRNA vaccine adverse events is crucial.

In the context of tumors, cross-presentation can sometimes lead to limited T cell activation, albeit without inducing tolerance. This is possible because cancer cells can also manipulate cross-priming mechanisms to evade immune detection [84]. Remarkably, as noted above, cancer-associated fibroblasts can process and cross-present tumor antigens to CD8+ T cells, leading to antigen-specific T cell death and dysfunction [64,66]. Thus, the cross-presentation capacity of mRNA vaccines may be particularly deleterious in an immunocompromised or cancer environment.

### 5.3. A Novel mRNA Platform Facilitates Cell Surface Translocation—Unexplained T Cell Responses Congruent with Antigen Cross-Presentation

The disparate impact of antigen subcellular localization was very recently highlighted in a study that developed a novel platform involving chimeric antigens [10]. The authors emphasize the difference between mRNA technologies and traditional vaccines, which deliver antigens directly to the extracellular space. For mRNA vaccines, they confirmed the same problem first identified by Käemmerer et al. [38]. Some of the encoded proteins were retained in the cytosol rather than presented at the cell surface. Due to the difficulty for B cells and APCs to recognize these intracellular antigens, they were indeed associated with an insufficient immune response.

To enhance immune responses, cell surface translocation (CST) signals were grafted onto mRNA antigens and tested in mice. Indeed, this substantially increased antigen surface expression levels. Interestingly, the study revealed a positive correlation between the chimeric antigen surface expression and antibody response. However, when T cells were collected on day 28 from mice vaccinated with the modified mRNA vaccines, enhancing CST altered T cell responses in less predictable ways.

- In contrast to B cell responses, there was no obvious correlation between T cell response and antigen surface expression levels.
- This confirms that T cell responses are influenced by factors beyond just surface antigen expression levels and validates the key role of additional signals.
- Indeed, the chimeric CST antigens altered the T cell response in both ways, leading to either a substantially increased or a distinctively reduced T cell response.

The authors did not address this. However, these findings may, at least in part, be explained by the above:

- Enhanced CTL responses could have been facilitated by antigen cross-presentation in the context of appropriate signal 2 and 3 activation. For example, the new platform in [10] increased T cell responses to some chimeric E6 and E7 LNP mRNA, which are soluble antigens. For these, they observed higher activation-induced markers (AIMs) compared with control groups, suggesting an improved CTL response. Even though the authors did not analyze antigen cross-presentation, it is reasonable to expect that the induction of soluble antigens via CST contributed to antigen cross-presentation (pathway (d) in Figure 2). In the context of sufficient co-stimulation, as indicated in [10], this would engender potent CTL responses.
- On the other hand, the MVP modules mostly lowered the T cell response for M1R, an mpox virus (MPXV) type I antigen, which is expected to remain membrane-bound. If the vaccines mostly transfected non-immune cells, this could reflect reduced antigen cross-presentation for membrane-bound antigens, in line with what was observed in [26] for DNA vaccines.
- Some experiments in [10] also revealed an upregulation of CD25+ T cells, also for antigens with strong/improved membrane trafficking of mRNA vaccine antigens. Importantly, CD25 serves as a marker of both T cell activation and immune tolerance depending on the T cell context [85]. This further highlights the complexity of signals beyond T cell activation required to induce immunity or tolerance.

In conclusion, the disparate patterns identified in [10] align with key aspects of antigen cross-presentation. As suggested above, vaccine antigens that remain bound to non-APCs cannot effectively prime cellular responses. On the other hand, the addition of the CST signal to enhance the translocation of the encoded antigens may, in some cases, facilitate their secretion and propensity for antigen cross-presentation. But, again, the effect (diminished or enhanced immunity) would be further dictated by the type and degree of co-stimulatory and other signals. Without adequate activation of APCs, cross-presentation engenders a tolerogenic rather than an immune response. In such circumstances, even the optimized cell surface expression of the produced antigen, despite increasing specific antibody titers [10], would engender cellular tolerance.

## 6. Non-Specific, Heterologous, Off-Target Effects

Generally, specificity is the immune response’s ability to distinguish between different antigenic variants [86]. Antigen specificity is commonly understood in immunology as the immune system’s ability to mount a response against a particular, unique antigen or epitope and discriminate it from other antigens. However, antigen specificity does not imply that the entire immune response targets only one antigen exclusively.

A common phenomenon in immunology is cross-reactivity, whereby immune components (e.g., antibodies and T cell receptors) generated against one antigen recognize and react with different, often structurally similar, but distinct antigens that are not intentionally targeted by a vaccine. SARS-CoV-2 mRNA vaccines have been shown to elicit cross-reactive antibodies targeting both SARS-CoV-2 variants and certain seasonal human coronaviruses [87,88].

This section proposes off-target immune effects after mRNA vaccination that are not due to cross-reactivity. As such, mRNA vaccines may induce broader immune system modulation that affects unrelated antigens, pathogens, or immune responses. It will be described how and why such non-specific effects may emerge for pro-drugs and, in particular, in the context of antigen cross-presentation. Heterologous effects following mRNA vaccination have been described in the literature. It has been impossible to understand how these were initiated, also because of the disparity reported by different studies. The mechanisms described below will offer a potential explanation for these unresolved questions.

### 6.1. Immunostimulatory Components Are Required to Elicit Robust Antigen-Specific T Cell Proliferation—The Context of a Pro-Drug

In the naive state, CD8+ T cells of any particular specificity are typically present at very low frequencies. These cells are also initially in a quiescent state [48]. During the initiation phase, resting CD8+ T cells are activated to proliferate clonally and acquire effector functions. The processes involved are intricate. As noted above, generating a CD8+ T cell response requires antigen presentation on MHC-I molecules by APCs in the context of additional molecular cues (e.g., co-stimulation and cytokines) that drive T cell expansion and effector differentiation.

For vaccine design, the involvement of immune-stimulating adjuvants is pivotal to facilitate these cues [59]. Enormous efforts have been devoted to ensuring specific activation of T cells, afforded by such adjuvants. However, the situation of mRNA vaccines is entirely different.

mRNA vaccines are unique in that they function as pro-drugs [89]. In contrast to traditional vaccines, the administered mRNA itself is not directly pharmacologically active. Rather, these technologies rely on a complex process during which cells translate the synthetic mRNA into a protein antigen.

However, the immunological driver of immunity is not the protein product per se. Notably, both the mRNA and the LNP components have been found to inherently act as adjuvants, which, combined with the produced protein, engender immune responses [6,35]. However, how this may impact specific T cell immunity in this context of a pro-drug has not been elucidated.

**Figure 4:**
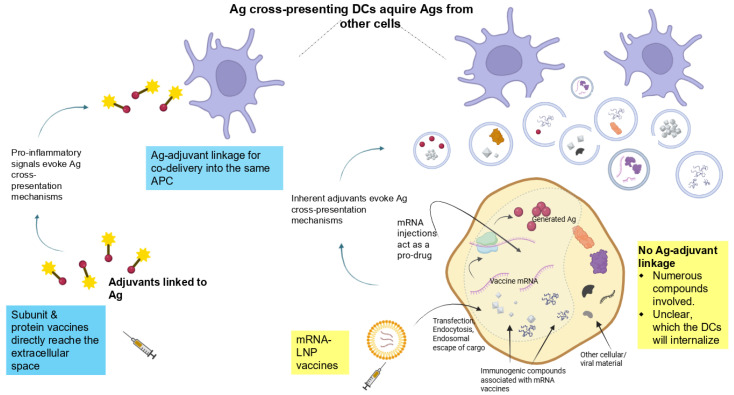
Summary of antigen cross-presentation in traditional versus mRNA vaccines: whereas subunit- and protein-based vaccines rely on directly linking adjuvants to the target antigen to facilitate specific immune responses, this approach does not extend to pro-drug platforms, such as mRNA vaccines. Created in BioRender. Mueller, S. (2025) https://BioRender.com/v52b520 (accessed on 22 September 2025).

Traditional vaccines crucially rely on cross-presentation of an exogenous antigen on MHC class I for generating a CD8+ T cell response. Above, arguments were provided for why this process may also be essential for mRNA vaccines. The key distinction from subunit, recombinant protein, or peptide vaccines is that, for these, the antigen can be precisely linked to an immune-stimulating adjuvant [59]. This decisive step to ensure a specific immune response cannot be supported in the same manner by a pro-drug. However, as illustrated in Figure 4, the intrinsic properties of mRNA technology limit the direct conjugation of an antigen and adjuvant required to prime resting CD8+ T cells toward a targeted antigen.

### 6.2. Insights About Early Immune Responses After mRNA Vaccines Confirm the Central Role of Adjuvants in Cellular Immunity

Research on early immune activation by mRNA vaccines highlights the critical role of adjuvants in shaping both general immune protection and specific CD8+ T cell responses.

Two studies [35,43] investigating the early immune response to mRNA vaccines report that type I interferon production is crucial for inducing antigen-specific cellular immunity in mice. Concretely, Kim et al. [35] found that IFN-β was expressed in injection site fibroblasts within about 2 h after the injection, specifically in response to the mRNA component, not to the LNP component of mRNA vaccines. In turn, type I IFN responses in migratory DCs were also specifically induced by the mRNA component. Additionally, the LNP component induced stromal pro-inflammatory responses. Yet, overall, the injection site IFN-β was suggested to guide the specific cellular immune responses against mRNA vaccines.

Notably, these early immune responses, which lead to antigen-specific cellular immunity, are driven by the vaccine’s components, not necessarily the resulting protein antigen. Even though Kim et al. frequently use the notation spike+ or spike-, in their study, they commonly refer to cells that do or do not harbor the vaccine mRNA, respectively, and not the encoded spike protein. Kim et al. demonstrate that early inflammatory responses at the injection site, triggered by the mRNA component acting as an adjuvant, are critical for inducing vaccine antigen-specific cellular immunity.

The analogous question is which factors govern the activation of specific T cell responses during antigen cross-presentation. Now, as indicated, the gene enrichment analysis in [35] identified this process to be dominant in the Fib_Cxcl5 population. In this case, Kim et al. suggest that the induction of these inflammatory fibroblasts at the injection site depends on the LNP component of the vaccine. Notwithstanding this, they find they respond to the vaccine mRNA as well. Specifically, the IFN beta gene, *Ifnb1,* was exclusively expressed 2h post-mRNA vaccine injection in fibroblasts positive for the vaccine mRNA, but not in empty LNP-injected fibroblasts. Therefore, these fibroblasts may be driven by both the LNP and mRNA components. As before, regardless of whether the injection site fibroblasts respond to the vaccine LNP, mRNA, or both, neither of them is the actual vaccine antigen. Instead, cellular immunity seems to be driven by adjuvants inherent in this platform. Particularly for antigen cross-presentation, these adjuvants may play an unanticipated role, undermining the specificity of the cellular immune response.

### 6.3. With mRNA Vaccines, Antigen Cross-Presentation May Enable Off-Target and Heterologous Effects

#### 6.3.1. Unique Considerations for mRNA Vaccines

The situation of mRNA vaccines is unique. These platforms encompass various immunologic compounds that could be internalized by immune cells. Specifically, during antigen cross-presentation, the nature of the transferred antigenic material cannot be predetermined. Due to the pro-drug nature of these platforms, the precise presence and concentration of vaccine components within a cell cannot be exactly defined. Sometimes, a transfected cell may contain many immunogenic compounds but only a few encoded antigens. Likewise, the various antigenic transfer mechanisms (Figure 2) cannot be limited to the encoded antigen alone. Besides these, receiving APCs may acquire LNPs, full-length or fragmented synthetic mRNAs, residual levels of dsRNAs, RNA:DNA hybrids, other manufacturing byproducts, or degradative products [6,38,90,91]. Possibly worse yet, a transfected cell will also contain endogenous or pathogenic proteins and other more or less immunogenic material.

Again, these aspects of antigen cross-presentation are entirely different than those of subunit- or protein-based vaccines, where the potentiation of an antigen-specific CD8+ T cell response is harnessed via adjuvants linked to the targeted protein or peptide antigen (Figure 4). The following section will propose why this provides the basis for undefined, off-target cellular responses.

#### 6.3.2. The Potentiation of Off-Target Antigen-Specific CD8+ T Cell Responses

In general, antigen transfer may entail an inoculated protein (such as in a vaccine), proteins from a circulating pathogen, or proteins released by cells (such as during an infection), involving the whole protein, fragments thereof, or complexes with heat shock proteins, apoptotic bodies from dying cells, and others [44].

**Figure 5:**
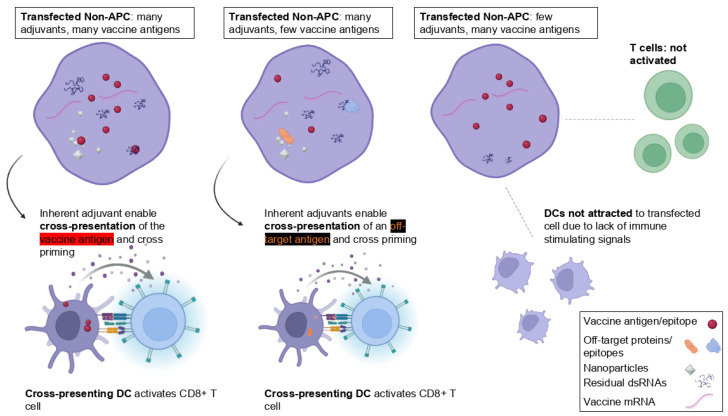
Dilemma of mRNA vaccine transfection of non-professional APCs: these cells poorly activate T cell immunity; nonetheless, intrinsic antigen cross-presentation processes can result in both antigen-specific and off-target CD8+ T cell activation. Created in BioRender. Mueller, S. (2025) https://BioRender.com/v52b520 (accessed on 22 September 2025).

This could impact the specificity of the immune response. However, this does not seem to have been analyzed from the perspective of pro-drugs. Particularly in the context of antigen cross-presentation, it seems unlikely that heterologous and off-target effects can be ruled out. This is because this process efficiently promotes antigen presentation on MHC-I to potentiate antigen-specific CD8+ T cell response. However, as just highlighted, this can likely not be limited to the targeted antigen alone (summarized in Figure 5).

#### 6.3.3. Off-Target Cross-Priming vs. Cross-Tolerance

Granted, it is possible that the antigenic components acquired by the cross-presenting APCs encompass pathogenic antigens, e.g., from latent or ongoing infections. The cross-presentation of epitopes from heterologous infections may be considered an inherent advantage that broadens the immune response. Nonetheless, in this context, it appears that the induction of either cross-priming or cross-tolerance cannot be controlled.

On the one hand, off-target cross-priming can lead to immune system overactivation to heterologous infections, cytotoxicity in normal tissues, uncontrolled inflammation, or autoimmune reactions. On the other hand, mRNA vaccine-generated cross-tolerance may not be restricted to self- or innocuous antigens (Figure 5). It is uncertain whether tolerance induction in this context could also apply to pathogenic agents or tumor cells (Figure 4).

### 6.4. Specific vs. Off-Target Immune Responses with Licensed mRNA Vaccines

Numerous studies have reported the specificity of mRNA vaccine-instilled immunity. These seem to contradict the potential for the postulated heterologous effects. However, the above mechanisms make it clear that antigen cross-presentation of pro-drugs could engage both specific and non-specific responses.

The ability of mRNA vaccines to generate antigen-specific CD8+ T cell responses has been thoroughly documented [92,93,94]. Yet, many of the issues described above were not foreseen during development and did not appear to be a primary focus at the time. For example, in their Study R-20-0072, Pfizer claimed to have proved the specificity of T cell activation. Nevertheless, this was only performed by examining reactivity towards AH1, an immunodominant tumor antigen. However, the absence of a T cell response to AH1 only means there is no response to that particular model antigen. It does not exclude that the vaccine could, via other mechanisms, induce responses to self-antigens or antigens from latent infections present in the host. Explicitly testing for or ruling out T cell activation against all possible epitopes from unrelated pathogens and self-antigens is impractical due to the vast number of potential antigens.

Despite this, significant efforts have been invested in recent years to understand the specificity of CD8+ T cell immunity induced by mRNA vaccines. Table 3 summarizes three excellent studies that aim to prove specificity and the absence of non-specific responses. Despite the technical progress made, the table highlights the complexity and limits of such an endeavor. Therefore, ruling out non-specific responses remains a major technical bottleneck.

Even so, it has also been confirmed that mRNA injections can modulate the innate immune responses to heterologous infections. This has been known since the seminal work by Föhse et al. [1], who demonstrated that the jabs may lead to altered immune defense against other pathogens and invaders than those targeted. This consequential insight has triggered intense research aimed at confirming whether the mRNA injections can engender responses against unrelated infections.

Meanwhile, several studies have confirmed heterologous effects on innate immune cells (Table 4). It has been suggested these are reminiscent of the induction of anti-inflammatory trained immunity (immune tolerance), facilitated through metabolic and epigenetic changes, which may even be inherited [2]. Off-target T cell immunity following mRNA injection is also believed to result from bystander activation, which aligns with observations of reactivation of unrelated pathogens in some patients after mRNA vaccination [95]. Still, these considerations have not been validated.

The non-specific effects are likely supported by several mechanisms and extend to other vaccine platforms. Specifically, for vaccines against SARS-CoV-2, both ChAdOx1-S (Oxford-AstraZeneca) and modified mRNA BNT162b2 (Pfizer-BioNTech) modulated immune responses to unrelated pathogens [3]. After the second vaccine dose, BNT162b2 recipients exhibited greater specific and off-target cytokine responses than ChAdOx1-S recipients. Still, the underlying mechanisms could not be identified.

From the perspective of genetic vaccines, because the encoded antigen(s) are clearly specified, non-specific responses seem difficult to explain. The notion developed above gives a rational explanation of why and how this could have unfolded. Table 5 provides a concise summary of the key arguments explaining why mRNA vaccines are believed to trigger non-specific immune reactions.

## 7. Discussion and Open Questions

This article discussed specific aspects of CD8+ T cell activation after i.m. mRNA vaccination. It is widely assumed that this is facilitated by direct transfection of APCs and engagement of the classical MHC-I pathway for endogenous antigens. However, several inconsistencies and challenges after i.m. mRNA-LNP administration include the following:

- Transfection may occur broadly in tissue cells rather than being restricted to professional APCs only. Yet, somatic cells lack the full machinery necessary for complete cellular immune induction.
- It has been shown that T cells are largely absent at the injection site [28]. By contrast, T lymphocytes primarily first encounter antigens and undergo effective activation in the secondary lymphoid organs.
- For a potent activation of CD8+ T cells, the immunogenic material of mRNA injections must be adequately presented by professional APCs, e.g., in the LN T cell zone, along with adequate co-stimulation.

**Figure 6:**
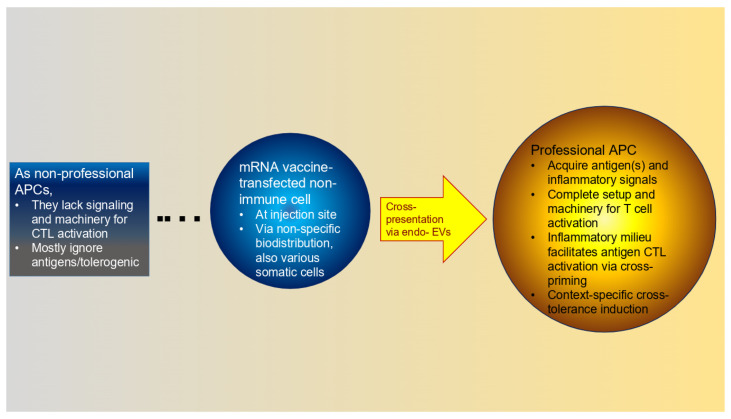
Antigen cross-presentation may facilitate CTL activation, even when mRNA vaccines transfect somatic cells. Many cells that are transfected are non-professional APCs and do not activate cellular immunity. However, CD8+ T cells can be activated in an indirect process involving antigen cross-presentation. Various antigenic components, transmitted from transfected somatic cells to professional APCs, when supported by appropriate activation signals, can effectively cross-prime CTLs. However, in a context of insufficient co-stimulation, this can also lead to cross-tolerance.

The process of antigen cross-presentation may emerge naturally, potentially offering a solution to the above (Figure 6). In fact, in this context of transfection of non-immune cells, this process may be essential. First, via transfer of the antigenic material, this process provides the potential for effective T cell responses via professional immune cells. Second, without this step, i.m. injection results in the risk of automatic tolerance induction; indeed, lacking the important machinery and signaling, non-APCs, when transfected, may, instead of immune activation, engender tolerogenic effects or ignore the novel antigen produced by the injections. Nonetheless, cross-presenting APCs, without appropriate activation and survival signals, may still trigger tolerance.

A concise summary of the potential benefits and overlooked concerns of antigen cross-presentation following intramuscular mRNA vaccination is given in Table 6. The above considerations raise numerous new open questions. Some will be highlighted in the following.

### 7.1. Antigen Cross-Presentation for mRNA Vaccines, an Unrecognized Potential at the Price of Off-Target Effects

Apart from direct APC transfection, antigen cross-presentation is the sole mechanism for eliciting effective CTL responses with mRNA vaccines. Despite this potentially enormous benefit, this process has been underappreciated for mRNA vaccines. The review above identified very few studies that considered this pathway in this context. According to the above rationale, it may play an essential role.

Despite its name, for mRNA vaccines, antigen cross-presentation may or may not involve the antigen encoded by the platform (Figure 5). Because the targeted antigen is processed inside a cell and the mRNA-LNP injections inherently encompass several immunogenic compounds, it is impossible to limit the transfer of antigenic material from the transfected somatic cells to the receiving APCs. As a result, the cross-presenting immune cells can acquire various combinations of (self)antigens besides the one encoded, along with other compounds with undefined immunogenic potentials. This unfortunate situation is the basis for enabling off-target, non-specific innate immune effects. These responses, originating from their pro-drug nature, are profoundly different from mRNA vaccines, which instill cross-immunity.

The impossibility of linking the vaccine antigen to an agent with adjuvant properties in a pro-drug context does not seem to have been described before. It is unknown to what extent this could provoke off-target immune responses even when APCs are directly transfected. With cross-presentation, this concern is greatly amplified.

The real possibility of undefined antigenic transfer challenges the notion that the cellular response can only be towards the targeted antigen alone. At the same time, as cross-presentation pathways unfold, this may also result in (cross)-tolerance. It is not known how to ensure the requirement for appropriate pro-inflammatory signals. While mRNA-vaccinated individuals may suffer adverse side effects from off-target cytotoxic reactions, they might also experience reduced protection against SARS-CoV-2 or other infections, including those to which they were previously immune.

### 7.2. Could Antigen Cross-Presentation Be Harnessed, Akin to Protein and Subunit Vaccines?

Off-target cross-priming/cross-tolerance may engender considerable adverse effects. Considering the potential benefits of antigen cross-presentation (Table 7), one may ask if the undesirable outcomes could be circumvented. Extensive research has explored methods to harness cross-presentation for vaccines. These approaches all critically rely on the molecular targeting of professional APCs, such as DCs, and on mechanisms to support antigen release (Figure 3) from endosomes into the cytosol [98]. For a specific CD8+ T cell response, they require appropriate adjuvants. Furthermore, more traditional vaccines inject their material directly into the extracellular space, making it more accessible to immune cells. Therefore, these considerations have not been developed for mRNA platforms, which express the antigen intracellularly and which inherently resemble pro-drugs.

Table 8 compiles some of the profound obstacles encountered for mRNA vaccines when trying to extend the approaches developed for other vaccine technologies to facilitate specific cellular responses.

### 7.3. Why mRNA Vaccines Are Likely to Naturally Engage Cross-Presentation

For mRNA vaccines, a technical solution for targeting antigen cross-presentation to potentiate the targeted antigen alone will require a completely different approach. Even if that were possible, this would likely not solve the potential for the unwanted effects highlighted above. The reason for this is that the process of antigen cross-presentation cannot be avoided unless the injections exclusively transfect immune cells, which seems unlikely.

Now, as soon as the product transfects non-APCs, this will start various natural mechanisms to trigger cross-presentation (Figure 2). Notably, as indicated before (Figure 3), mRNA vaccines may even more readily engage endosomal escape and other steps known to be bottlenecks for antigen cross-presentation in more traditional vaccines. The natural activation of these mechanisms with mRNA vaccines is supported by the following:

- Cross-presentation is specifically known to be activated by interferons [104,105], a hallmark of the mRNA vaccine immune response [35,43]. The injections induce a localized inflammatory response that attracts immune cells, which, based on the compounds’ characteristics such as type, size, and accessibility, engulf them through phagocytosis or endocytosis.
- Exosomes may be triggered by the inflammatory mRNA vaccine compounds [31,35] or their toxic effects facilitated by the LNPs [32,33,106] and during the transfection process itself, which has been shown to engender substantial cell health impairment [38]. Notably, exosomes are known to be released into circulation in response to various environmental stimuli, including drugs and toxic agents, nanoparticles, inflammation, systemic immune responses, oxidative stress, cellular stress, and cell damage [54,107,108]. Importantly, this means they may not merely disseminate protein antigens but also biological activities, including immune-stimulatory signals.
- Very recent research has highlighted the importance of cell surface translocation of the encoded antigens, which has been targeted via the incorporation of specific signaling sequences [10]. In turn, if these modifications cause fragments of the antigens to be released from the cell membrane, this may further enhance undefined antigen cross-presentation.

### 7.4. Durability and Scope of the Response and Factors That Drive Tolerance

It is generally assumed that rapid antigen degradation destroys many epitopes before they can be adequately processed and presented on MHC-I molecules [53]. Nonetheless, some cross-presentation mechanisms may regulate antigen degradation rates within APCs, resulting in diminished antigen degradation [53]. This can allow prolonged availability of epitopes on MHC I, which is essential for sustained T cell activation after migration to lymph nodes. For mRNA vaccines, the inherently prolonged presence of the antigen(s) produced by these injections may additionally facilitate the accumulation and availability of antigens for antigen cross-presentation. Differences compared to other platforms have not been investigated.

Likewise, the durability of various cross-primed cytotoxic responses may not be easily predictable, as this is intimately linked to the presence of appropriate co-stimulatory and survival signals. As such, tolerance emerges as a plausible feasibility. It is unclear how this relates to characterized mechanisms in how mRNA vaccines lead to diminished immunity and tolerance [9,79,109,110]. It is also unclear if ongoing off-target cross-presentation could influence existing immunity. For example, DC-mediated cross-presentation is thought to contribute to the reactivation and expansion of CD8+ T cell populations in HIV infection, even though continuous exposure to antigens is also known to weaken responses [111]. A critical concern with mRNA platforms is that undefined antigen cross-presentation may interfere with immune imprinting, potentially weakening pre-existing immune memory from prior infections or vaccinations and diverting cellular responses toward unintended antigens.

### 7.5. Systemic and Environmental Effects

The danger of systemic reactions following mRNA injections and their ensuing deleterious effects is undisputed [2,31,32,33,112,113]. This has triggered intense research to develop mRNA technologies with more predictable and limited biodistribution and persistence. Still, the above has shown why these efforts may have limited success. Regardless of any limited LNP carrier biodistribution or less inflammatory lipids, this does not resolve the fact that both the vaccine LNPs and mRNA components can act as adjuvants [35].

One of the key challenges is that an effective CD8+ T cell response depends on the presence of suitable adjuvants or a proinflammatory environment; without these, immune tolerance is likely to develop. While this decision is critical, the behavior and distribution of immunogenic agents in pro-drugs are not well characterized. The concern over adverse systemic effects is amplified by novel platforms such as that of Aunins et al. [11], who introduce a potent adjuvant separately to enhance CD8+ T cell activation, thereby adding a complex layer to the widely overlooked pro-drug characteristics inherent in the platform.

Antigenic transfer from transfected non-immune cells is a natural phenomenon. The extent to which this process, unwittingly induced by mRNA vaccines, may or may not run off is unknown. For example, biological carriers, such as endogenous EVs [114], may disseminate the engendered products, immune activation states, and biological activities systemically [114] or even to close contact organisms [115]. As such, cross-presentation via EVs may account for some of the observed immune reactions in close contacts/negative controls [37]. Yet, to what degree the transfer of synthetic genetic compounds and their induced activities could result in biological consequences in exposed organisms throughout the biosphere has not been studied [115].

## 8. Conclusions

**Figure 7:**
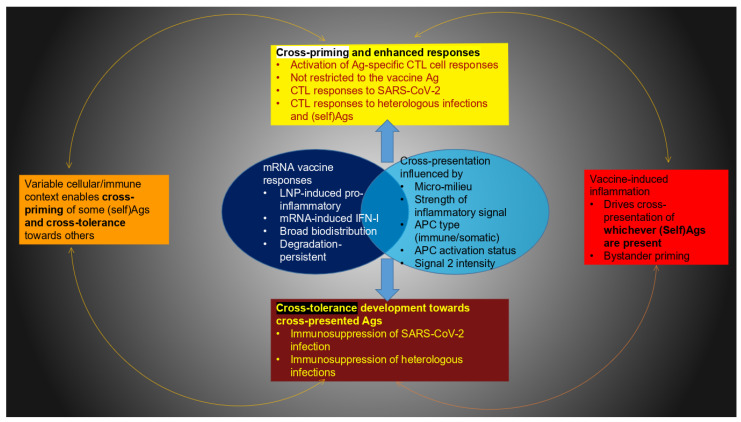
Mechanisms and effects of antigen cross-presentation facilitated by mRNA vaccines. This study described key mechanisms to explain the potential of mRNA vaccine cross-priming and cross-tolerance development, not only towards the vaccine-induced antigen but also unintended (self)antigens. These processes, naturally evoked in various contexts, likely also shape mRNA vaccine immunity. Although widely unappreciated, they may help resolve some of the paradoxes surrounding this platform. But their involvement may also have far-reaching implications and raise numerous open questions.

This study, based on an extensive literature review and rational analysis, provided the first comprehensive depiction of the benefits and concerns related to antigen cross-presentation of mRNA vaccines (summarized in Figure 7). It presents arguments supporting the idea that this natural T cell activation process, which has been largely overlooked in the context of mRNA vaccines, may represent a crucial missing element in understanding the cellular immune responses elicited by this platform. Key questions for future investigation were highlighted.

The analysis revealed multiple underrecognized factors contributing to the challenges in achieving robust CD8+ T cell activation following intramuscular mRNA vaccination. Intriguingly, with more traditional platforms, the induction of potent CTL responses is being targeted via antigen cross-presentation processes. Despite its numerous benefits, this process is still largely ignored for mRNA vaccines. Arguments were provided as to why, in this context, harnessing and controlling it may prove unfeasible.

Nonetheless, this alternative T cell activation mechanism can unwittingly be induced by natural processes. Although a limited number of mRNA vaccine studies have demonstrated the critical role of antigen cross-presentation in inducing T cell immunity, this pivotal aspect has remained largely overlooked. The fundamental mechanisms, including those that are triggered naturally, remain insufficiently explored.

mRNA vaccines, as pro-drugs, raise the possibility for both specific and non-specific CD8+ T cell responses, leading to undefined off-target effects. Moreover, the inherent adjuvanticity of this platform, a key determinant in this process, although known to be pro-inflammatory, is not enough to dictate its outcome. In the absence of complete activation of cross-presenting antigen-presenting cells, inflammatory signals alone may still induce immune tolerance instead of protective immunity.

Although the above observations offer a sound rationale for some of the mysteries and open questions related to the mRNA injections, they raise significant concerns. In some recipients, unintended immune reactions may become excessive, damaging healthy cells or tissues, triggering or worsening autoimmune responses, and, influenced by various identified factors, potentially promoting tumor development or the establishment of immune tolerance. A critical unresolved question is whether cross-priming triggered by mRNA vaccines can overcome immune memory against unrelated pathogens, or whether cross-tolerance might suppress pre-existing immunity. The identified immune-activating and -tolerizing mechanisms may have significant environmental implications, supporting and broadening current concerns regarding the role of exosomes in both established and emerging mRNA technologies across human, animal, and wildlife contexts [115].

In light of the numerous adverse effects reported among mRNA vaccine recipients, coupled with the resurgence of infectious diseases and compromised immunity, a clear understanding and resolution of the identified challenges are paramount for the broad application of these technologies in humans and animals towards existing and emerging pathogens.

## Figures and Tables

**Table 1:** Articles identified in PubMed about “antigen cross-presentation mRNA vaccine,” ignoring those that deal with “antigen presentation” and non-mRNA vaccine platforms (as of 27 August 2025).

Article Title and Reference	Comment
“Rapid Surface Display of mRNA Antigens by Bacteria-Derived Outer Membrane Vesicles for a Personalized Tumor Vaccine,” [14]	This article mentions “cross-presentation via listeriolysin O-mediated endosomal escape.” However, the proposed platform provides a delivery technology distinct from lipid nanoparticles and, therefore, cannot directly inform about mRNA-LNP vaccines.
“SMART-lipid nanoparticles enabled mRNA vaccine elicits cross-reactive humoral responses against the omicron sub-variants,” [15]	This article deals with aspects of cross-reactive vaccines but does not cover antigen cross-presentation.
“Efficient Nanovaccine Delivery in Cancer Immunotherapy,” [16]	This article also utilizes a different platform from LNPs. Rather, the authors discuss nanovaccine engineering to codeliver adjuvants and multiepitope antigens into lymphoid organs and into antigen-presenting cells to facilitate cross-presentation of antigens. This relies on an entirely different delivery method than that utilized in the mRNA-LNP platforms.
“Soluble-microneedle enhance three T-cell activation signals as efficient tumor vaccines for melanoma prevention and treatment,” [17]	The DOTAP with a positive charge can electrostatically adsorb IL-12 mRNA with a negative charge and promote the transfection of IL-12 mRNA in DCs. Again, this proposes a novel delivery and DC-targeting method. The article does not provide information about antigen cross-presentation of existing mRNA vaccines.
“Longitudinal Analysis of Coronavirus-Neutralizing Activity in COVID-19 Patients,” [18]	Even though the article uses the term “cross-priming,” here, it is exclusively in the context of cross-reactive immune responses against unrelated antigens, including cross-booster effects. The article does not discuss antigen cross-presentation per se.
“Role of aptamer technology in extracellular vesicle biology and therapeutic applications,” [19]	Here, the authors rely on aptamers, synthetic nucleic acid molecules, to bind targets with high sensitivity and specificity. The aptamer technology discussed in the article serves as a tool for EV targeting and engineering. Again, this is a different technology from that employed by the mRNA-LNP platforms. Although antigen cross-presentation is mentioned, it is only briefly mentioned as a motivation for EV-based therapeutics. Antigen cross-presentation is one of their clinical applications that has been researched extensively. Specifically, mRNA vaccines are only mentioned as a new frontier for EV-based therapy. The article does not discuss antigen cross-presentation related to mRNA vaccines.
“Antigen-specific T helper cells and cytokine profiles predict intensity and longevity of cellular and humoral responses to SARS-CoV-2 booster vaccination,” [20]	The study examines cross-reactive T cells after primary mRNA vaccination against SARS-CoV-2. However, it does not discuss antigen cross-presentation per se.
“CD8 T-cell priming upon mRNA vaccination is restricted to bone-marrow-derived antigen-presenting cells and may involve antigen transfer from myocytes,” [21]	This 10-year-old article proposes that upon vaccination with self-amplifying mRNAs (SAMs), the antigen is expressed within muscle cells and then transferred to APCs. This indeed suggests that with SAM platforms, cross-priming is the prevalent mechanism for priming the CD8 T cell response. This technology uses a SAM platform based on an alphavirus genome. This contains genes encoding the viral replicase complex to enable amplification of the RNA, but lacks the genes encoding the viral structural proteins required to produce infectious viral particles. The study was unable to describe the cellular mechanism by which cross-priming occurs in vivo.
“Co-administration of GM-CSF expressing RNA is a powerful tool to enhance potency of SAM-based vaccines,” [22]	The study only briefly mentions antigen cross-presentation by referring to the earlier work [21] about SAM-based vaccines. Commenting on prior work, the authors of [22] reiterate the main findings made in [21] (see also Table 2). SAM technologies mostly transfect myocytes at the inoculation site. Despite this, CD8+ T cell priming is initiated by professional APCs rather than myocytes. APCs are barely found in the normal muscle tissue. However, they can migrate to the site of inoculation in response to inflammatory or chemotactic signals. As a result, infiltrating APCs can present the SAM-expressed antigen to T cells directly or through cross-presentation. The latter, they believe, works following the apoptosis of transfected myocytes and the consequent release of the antigen-associated apoptotic bodies, which, in turn, get phagocytosed by APCs and presented via the MHC class I.

**Table 2:** Antigen cross-presentation—feasibility and considerations for mRNA vaccines based on early insights about SAM platforms.

Key Attribute of SAM Antigen Cross-Presentation, as Determined in [21,22]	Analogs/Differences for Non-SAM Platforms
In mice injected intramuscularly with different SAM platforms, antigen cross-presentation was found to be required for CD8 T cell priming [21]. Antigen expression occurred mostly in the muscle fibres. BM-derived APCs were able to acquire the antigen from transfected mycroblasts. CD8+ T cell activation was observed only when the APCs were BM-derived, not when restricted to muscle cells. This indicates that muscle cells do not prime CD8+ T cells. Additionally, direct transfection of APCs by SAM vaccines was not required for CD8 T cell priming in vivo. Indeed, the experiments in [21] indicate that the APCs tested (BM-DCs and primary DCs) could not be transfected by SAM vaccines administered intramuscularly.	Analogous experiments for non-SAM platforms do not seem to have been conducted yet.
For antigen cross-presentation, the following factors were regarded as instrumental to help attract APCs: Replication of transfected RNA; Antigen expression; Transfection itself.	With non-SAM technologies, the synthetic mRNA is not expected to be replicated in vivo, excluding this notion as a key contributor to immune cell attraction and activation. With currently licensed mRNA vaccines, antigen expression and their translocation to the cell surface are not readily guaranteed. Emerging research indicates that this is a general problem, where some antigens created by these technologies are unable to make it to the surface, evading the body’s immune response system [10]. This raises the risk for tolerance rather than immune induction. On the other hand, it is reasonable to assume that the transection process itself can trigger antigen cross-presentation. The attraction of immune cells has been demonstrated. Specifically, a study [35] that investigated the effect of mice challenged with LNP-mRNA determined that the LNP component of the vaccine triggers inflammatory fibroblasts at the injection site that express diverse chemokine genes and recruit immune cells. The study also found that mRNA-LNP, but not LNP alone, induces migratory DCs highly expressing IFN-stimulated genes, both at the injection site and draining LNs.
The role of dsRNAs: SAM platforms produce numerous double-stranded RNAs (dsRNAs) during RNA replication. dsRNAs act as potent stimulators of innate immunity, engendering enhanced immune responses [21]. In [21], BM-DCs not only acquired the antigen from transfected myoblasts, but they also acquired the dsRNAs from them.	In recent years, much effort has been placed on reducing dsRNAs from in vitro-based RNA technologies due to their propensity to cause aberrant or excessive immune responses. However, their inherent potent activity as stimulators of innate immunity makes them potent self-adjuvants in these mRNA vaccines. The agents driving innate responses necessary for the induction of antigen cross-presentation mechanisms do not seem to have been studied.
The role of apoptosis: The vaccine-engendered antigens, following apoptosis of transfected cells, lead to their phagocytosis by professional APCs.	Apoptosis could also destroy the vaccine mRNA and is, generally, not wanted for mRNA vaccines. Indeed, one of the key features of the Nobel-prized nucleoside base modifications by Karikó and Weissman [36] was precisely to “suppress the potential of RNA to activate DCs.” Nonetheless, mRNA vaccines can trigger the release of exosomes [37,38,39], which include apoptotic bodies. Antigen transfer from non-immune to immune cells comprises numerous mechanisms. Besides apoptosis, other possible modalities are envisioned in the text.
The need for innate immune stimulators: The study [21] utilized non-modified RNAs, which are inherently immunogenic. Nonetheless, they observed that chemical manipulation of the mRNA sequence can enhance their intrinsic capacity to stimulate the innate immune system [21]. Modification of their platform also involved the expression of additional molecules to stimulate immunity and/or act as co-stimulatory molecules [21]. In [22], the authors employed a SAM platform to express, besides a model antigen, additionally, the cytokine GM-CSF as an immunopotentiator. GM-CSF, acting as a vaccine adjuvant, has the ability to induce recruitment, maturation, and activation of APCs. GM-CSF promoted an increased recruitment of APCs at the site of injection and an effective antigen-specific CD8+ T cell response.	Combined with purification from in vitro RNA production, the nucleoside-modified mRNA components have even been referred to as immuno-silent [36,40,41,42]. Nonetheless, these platforms exhibit inherent adjuvanticity. Notably, the LNP component of mRNA vaccines, particularly the cationic (ionizable) lipids, is well-known for its toxic and pro-inflammatory effect [31,32,33]. Besides the LNPs, it is now confirmed that the mRNA component also inherently acts as an adjuvant [35,43]. Studies have been able to demonstrate strong type-I IFN induction post-SARS-CoV-2 mRNA vaccines [35,43], facilitating CD8+ T cell activation.

**Table 3:** Examples of some state-of-the-art studies demonstrating specific CD8+ T cell activation by mRNA vaccination, how this was validated, and why off-target effects could not be entirely ruled out.

Reference	How the Experiments Set Out to Prove Specific Responses	Comment
Ref. [94]	Spike-specific mobilization of CD8+ T cells by SARS-CoV-2 mRNA vaccine was confirmed by Using unvaccinated sample controls to provide a baseline and help determine detection limits. Specific peptides to stimulate and expand T cells in vitro for assessment of their effector function. Tracking spike-specific CD8+ T cells via tetramers designed to bind specifically to T cell receptors and examining their proliferation. Performing sequence homology analyses to confirm that the epitopes were unique to SARS-CoV-2 and not conserved in other coronaviruses.	The detailed analysis confirmed specificity. Epitopes are not highly conserved between SARS-CoV-2 and SARS-CoV-1, MERS, or common cold coronaviruses. This ruled out responses to other coronaviruses. However, this only showed that a specific response was generated to a targeted epitope. This analysis cannot rule out additional activation towards off-target epitopes, e.g., from unrelated infections.
Ref. [93]	The study found that the BNT162b2 mRNA vaccine induced antigen-specific but cross-reactive CD8+ T cells. This was carefully characterized as follows. Peripheral blood mononuclear cells (PBMCs) from 21 BNT162b2-vaccinated healthy donors were isolated and stimulated with WT, Delta, or Omicron strain-derived spike peptides. Antigen-specific T cells were identified by a significant increase in relevant activation markers after peptide exposure. The activated T cells were subjected to functional and molecular analyses. Activated CD8+ T cells responding to spike peptides were sorted for TCR sequencing. PBMCs were also incubated in medium alone (unstimulated control) to serve as a baseline for spontaneous activation during the experiments.	Isolation procedures typically remove plasma and serum proteins and contaminants. The donors in the study were also healthy. Because the study sorted CD8+ T cells based on activation marker expression induced by stimulation with SARS-CoV-2 spike peptides only, it highly enriches for spike-specific T cells rather than bystander or non-specific populations. This would have impacted the entire downstream analysis. The experimental setup, involving purification, cultivation times, peptide specificity, and gating strategies, inherently minimizes non-specific activation from unrelated specificities. Therefore, the unstimulated (medium-only) control cultures are not expected to contain bacterial proteins or natural pathogen components other than the synthetic spike peptides.
Ref. [92]	The study surprisingly observed a higher frequency of vaccine-induced antigen-specific CD8+ T cells in chronic lymphocytic leukemia (CLL) and myeloid dysplastic syndrome (MDS) patients than in healthy controls. The specificity of the vaccine-induced T cell activation and persistence of memory T cells was established via the following processes: DNA-barcoded peptide–MHC (pMHC) multimers were used that precisely target T cells recognizing specific SARS-CoV-2 spike protein epitopes. After staining PBMCs with these multimers, multimer-binding CD8+ T cells were identified and sorted. This precisely mapped vaccine-induced T cell specificities. Additionally, 67 peptides from cytomegalovirus (CMV), Epstein–Barr virus (EBV), and influenza (FLU) (together denoted as CEF) were included to compare spike to CEF-antigen-reactive T cells. The CEF analysis examined CD8+ T cell responses against spike and CEF peptides. CEF epitope-specific CD8+ T cells exhibited no changes pre- to post-SARS-CoV-2 vaccination, except for a rise in influenza epitope-specific CD8+ T cells in a few patients. This, the authors reasoned, likely resulted from influenza vaccination.	The experiments confirm that vaccination induces spike-specific responses. A small rise in influenza epitope-specific CD8+ T cells was observed in a few patients following BNT162b2 vaccination. This was postulated to have been caused by concurrent or prior influenza vaccination. Unfortunately, this was not confirmed. Cross-reactivity or non-specific activation from the COVID-19 mRNA vaccine was not explicitly analyzed, but could be an alternative explanation. For CLL patient samples, non-specific activation from unrelated pathogens was automatically minimized during PBMC isolation. For MDS patients and healthy cohort samples, PBMCs were used for T cell staining without isolation. Without knowing details, it is possible that the influenza epitope-specific CD8+ T cells originated from purified or unpurified PBMCs. The latter would be highly conducive to antigen cross-presentation. The finding shows the difficulty of attributing the causes/mechanisms without additional information and performing further analyses.

**Table 4:** Examples of heterologous effects triggered by mRNA vaccination.

Heterologous Effect	Comment
Föhse et al. [1] reported the following for vaccination with BNT162b2 in healthcare workers: It not only induces specific adaptive immunity against SARS-CoV-2 but also alters innate immune responses to heterologous (non-SARS-CoV-2) stimuli. After vaccination, immune cells show long-term transcriptional changes and a modulation of cytokine production. Specifically, in response to BNT162b2, the cytokine response upon stimulation with the bacterial pathogen *Staphylococcus aureus* and the fungal pathogen *Candida albicans* tended to increase six months after the first dose of BNT162b2.	The off-target effects align with antigen cross-presentation involving epitopes of common human pathogens.
Qin et al. [2] found the following after pre-exposure to mRNA-LNPs in mice: The resistance to *Candida albicans* decreased. The resistance to the influenza virus was enhanced. The synthetic ionizable lipid component of the LNP, responsible for vaccine inflammation, plays a key role in these heterologous effects that can involve long-lasting immune changes.	The effects are congruent with antigen cross-presentation if the tested mice had antigens to these pathogens. As highlighted in the text, adjuvants play a pivotal role in driving reactions towards whichever antigens are present. The disparate outcome (immunity vs. tolerance) between Föhse et al. and Qin et al. could be explained by differing co-stimulatory and survival signals.
Noé et al. [4] identified that the BNT162b2 vaccine induces off-target (heterologous) effects on the pediatric immune system, altering cytokine responses to a broad range of microbial and viral stimuli.	The heterologous stimulants comprised common bacterial species, opportunistic fungal pathogens, viral antigens from childhood vaccines, and some synthetic antigens. Among these, the former class of stimulants involved common antigens that could have been present from common infections. The latter group consisted of poly(I:C) and R848 (Resiquimod). These closely resemble double-stranded RNA and double-stranded RNA-like components of the mRNA vaccines. As residual byproducts, it is possible these could be engaged during the antigen-cross presentation process (Figure 4 and Figure 5).
Connolly et al. [5] also discovered non-specific effects of BNT162b2 in eight volunteers. These mostly involved decreased cytokine responses by innate immune cells in response to heterologous stimuli. Notably, they observed this tolerance-like effect already early in response to the primary vaccination.	These heterologous effects were seen in response to common bacterial, fungal, and viral stimulants post-vaccination. As before, this is congruent with antigen cross-tolerance.
A recent study [95] targeted SARS-CoV-2 non-structural proteins at 6 months post-second dose in cross-reactive participants. It identified surprising increases in non-spike-specific T cell responses in the majority of vaccine recipients assessed, particularly related to non-structural proteins (NSPs). The study also noted highly significant increases in T cell responses specific to unrelated antigens, including those of common viral pathogens.	As before, these non-specific effects are in line with antigen cross-presentation involving common antigens, including those from prior SARS-CoV-2 infection.

**Table 5:** A nutshell summary of why mRNA vaccines may engender indiscriminate activation of T cells, leading to non-specific, off-target effects.

General Crucial Aspects	Relevant Features of mRNA Vaccines
The goal of adjuvants is to activate APCs and increase MHC–peptide presentation (signal 1) and co-stimulatory signals plus inflammatory cytokine production (signal 2).	mRNA vaccines incorporate several compounds that inherently act as adjuvants.
Adjuvants create an immune-stimulating milieu that can simultaneously amplify responses to all antigens present in that environment [96], not just one specific antigen.	mRNA vaccines act as pro-drugs. As a result, the following occur: The kinetics and cellular localization of the encoded antigen do not necessarily overlap with those of its inherent immune-stimulatory compounds (Figure 4). The process of antigen cross-presentation further severs the link between the inherent adjuvants and the targeted antigen. The inherent adjuvants may co-localize with off-target antigens in (cross-presenting) APCs.
Adjuvants cannot discriminate which antigen to act upon. They simply create a more stimulatory environment [96] covering all antigens present.	As there is no inherent mechanism in adjuvants to “select” a specific antigen to amplify, the inherently immunogenic compounds of mRNA vaccines can act upon both the targeted and other antigens present. This concern may be further amplified during antigen cross-presentation (Figure 5). During this process, adjuvants can increase uptake and retention in cross-presenting APCs, enhance expression of co-stimulatory molecules, and enhance secretion of cytokines, which potentiates CD8+ T cell responses to any of their cognate antigens present.
More traditional vaccines often contain multiple antigens from different pathogens combined with one or more adjuvants [97]. This is intentionally carried out to engender immune responses simultaneously against several antigens.	mRNA vaccine platforms can include more than one mRNA sequence to target several epitopes simultaneously. However, such a targeted polyspecific response is entirely different from the non-specific activation, which is facilitated by the pro-drug nature of the platform. In an antigen-rich context, one cannot exclude broad potentiation of cellular immune responses for multiple antigens simultaneously. This case may give rise to specific and non-specific CD8 T cell activation.

**Table 6:** Take-home summary of the unique aspects of antigen cross-presentation with i.m. mRNA vaccines.

Main Point	Underappreciated Aspects
With i.m. mRNA vaccines, CTL induction may critically rely on antigen cross-presentation.	For SAM platforms, antigen cross-presentation is required for CTL responses. One study reported the essential role of antigen cross-presentation for CD8+ T cell activation after COVID-19 mRNA vaccination. Evidence has shown that direct antigen expression on APCs following i.m. administration may not enhance CD8 T cell responses compared to antigen cross-presentation.
The pathways and processes involved in antigen cross-presentation evoked by mRNA vaccines are not known.	Even though antigen cross-presentation has been implicated with current mRNA vaccines, no study has detailed the relevant underlying mechanisms. The triggers of mRNA vaccine antigen cross-presentation have not been described. It is unclear in which contexts which of the various antigen cross-presentation pathways are evoked. There is no understanding of how the different triggers and processes result in antigen cross-priming vs. cross-tolerance.
In contrast to mechanisms and pathways involved in antigen expression and presentation, those related to antigen cross-presentation are entirely different.	Notably, with antigen cross-presentation post-mRNA vaccines, the vaccine antigen is not the key agent eliciting CD8+ T cell response.
Some evidence suggests that, for mRNA vaccines, direct expression of the antigen in professional APCs may not be necessary for T cell immunity. I.m. mRNA vaccines often transfect non-immune cells at the injection site. Via “antigen transfer,” APCs can acquire various immunogenic compounds from these non-immune cells. These may or may not be limited to, or even include, the vaccine antigen.	Some COVID-19 mRNA vaccines have shown that, rather than the antigen, it is one of the following, or their combination, that activate APCs: LNP-mRNAs or the LNP component. For SAM platforms, it has been suggested that dsRNA byproducts are also able to attract and activate APCs. This suggests that other inherent immune-stimulating components (residual manufacturing byproducts) of non-SAM platforms can perform the same. Therefore, despite the name antigen cross-presentation, with mRNA vaccines, CD8 T cells may not be directly activated by the vaccine antigen. Rather, the activation may rely on the intrinsic adjuvanticity of the platform.
There is no guarantee that CD8 T cell priming is directed towards the vaccine antigen alone.	Due to their pro-drug nature, the localization/fate of inherent immunogenic compounds that activate CD8 T cells is disjoint from that of the vaccine antigen. Without a clear link between the vaccine antigen and an adjuvant, the priming of the CD8 T cell response by mRNA vaccines may not be predominantly towards the targeted antigen (if, at all). The inherent adjuvanticity of mRNA vaccines, in the context of the platform as a pro-drug, may enable the priming towards off-target (self)antigens.

**Table 7:** Known benefits of antigen cross-presentation and their potential benefits for i.m. mRNA vaccines.

General Benefits of Antigen Cross-Presentation	Potential Benefits for mRNA Vaccines
Antigen cross-presentation allows for the detection of antigens and initiation of CD8+ T cell responses against viruses, tumors, and intracellular pathogens that do not infect APCs directly. As a result, professional APCs can capture and process antigens from dead or dying cells and present them to CTLs, thereby broadening the range of antigens that can be presented to CTLs [23,99]. This effectively enables the immune system to detect and respond to infections or tumors occurring in non-APC cells [48].	The analogous potential benefits are expected for mRNA vaccines because of the following: The targeting and direct transfection of professional APCs remain a challenge. mRNA-LNPs, such as those against COVID-19, are delivered intramuscularly. However, skeletal muscle contains very few DCs [12,22,51]. In addition to their limited presence, i.m. administration also suffers from the poor mobilization capacity of tissue-resident DCs [51]. Non-APCs, when transfected, cannot activate CD8+ T cells. However, cross-presenting APCs can do so effectively in the context of appropriate activation and survival signals.
Cross-presentation is also critical for immune tolerance, preventing autoimmunity by presenting self or harmless antigens, especially under non-inflammatory conditions, thus leading to peripheral tolerance rather than activation [48]. In a natural context, T cells die or become anergic if they do not receive co-stimulation (signal 2) from professional APCs [100]. Analogously, cross-tolerance, which normally results in the peripheral deletion of autoreactive CTLs, has an obvious protective function in the case of antigen-specific tolerization of CD8+ T cells by APCs that cross-present self- or innocuous antigens.	Potentially, this could have a role in contexts where inducing or maintaining peripheral tolerance is desired, particularly in therapeutic settings targeting autoimmune diseases or other immune-mediated disorders.
Antigen cross-presentation is pivotal for traditional vaccines that target CD8+ T cell responses, such as viral or cancer vaccines.	Early studies involving SAM platforms suggest that, in this case, too, antigen cross-presentation is required. The direct transfection of APCs was difficult to achieve and not necessary for the induction of CD8+ T cell responses.

**Table 8:** The induction of antigen-specific CD8+ T cell responses with protein/subunit vaccines and mRNA vaccines; the differences and technical challenges have not been investigated. The table compiles key issues, based on an extrapolation of the expected relevant results available in the published literature.

Vaccines Based on Defined Antigen Subunits, Recombinant Proteins, and/or Peptides	Problematic Aspects When Trying to Extend These to mRNA Vaccines
Adjuvants are needed to [59,81,98]: Increase the weak capacity for protein- and peptide-based vaccines to stimulate CTL responses. Enhance expression of co-stimulatory molecules to avoid T cell tolerance due to antigen presentation by immature APCs. Induce the secretion of IFN-I and Th1 cytokines, to facilitate cross-presentation efficiency and lead to a more robust downstream CD8+ T cell response.	In contrast to more traditional vaccines, with pro-drugs, such as mRNA vaccines, the adjuvants cannot be explicitly linked to the targeted antigen (Figure 4). Adjuvanted mRNA-based vaccines, where adjuvants are integrated into the vaccine formulation, have been suggested to enable the co-expression of co-stimulatory molecules and cytokines. For example, CD40 ligand supplied as mRNA increased the anti-tumor effect of a two-component mRNA vaccine [101]. Whether such an approach can be modified for prophylactic vaccines is questionable. Balancing the requirement for a sufficiently large pro-inflammatory response (here, needed for T cell stimulation) whilst avoiding overreactions has proven difficult [32,33]. Additionally, i.m. vaccines, via their propensity for transfecting non-immune cells, risk the development of T cell tolerance due to antigen presentation by non-APCs [59]. Aside from explicitly integrating adjuvants into the vaccine formulation, the inherent adjuvanticity of the platform can cause problems. The type, quantity, and kinetics of immunogenic material acquired by cross-presenting APCs do not seem to be controllable (Figure 5). Aligning the requirements for effective CD8+ T cell activation is also difficult, as many of the mechanisms underlying antigen cross-presentation have not been studied for pro-drugs.
Concurrent signaling required to enable T cell responses: Naive T cells must recognize their cognate antigen via their T cell receptor, and, at the same time, they must receive co-stimulatory and survival signals [102]. To help facilitate this co-signaling, several strategies have been developed for coordinated codelivery of the antigen and adjuvant. These approaches aim to ensure the coordinated uptake of the antigen and adjuvant by the same APC.	Cross-presenting APCs can acquire various compounds inherent in mRNA technologies that act as adjuvants. Due to the pro-drug nature of these platforms, these are not automatically linked to the produced antigen. The vaccine antigens and inherent adjuvants can have disparate kinetics. Some of the inherent adjuvants may more readily be internalized by and activate APCs. For example, DCs can internalize nanoparticles ranging from 10 to 1000 nm more efficiently than a soluble antigen [59]. As a result, some APCs may acquire immunogenic compounds of the mRNA vaccines, such as LNP compounds, more readily than the vaccine antigen. This discord may support antigen cross-priming towards off-target antigens. Alternatively, some APCs may internalize the vaccine antigen without acquiring the appropriate co-stimulation, which leads to the concern of cross-tolerance induction. With mRNA vaccines, key aspects of this co-signaling requirement are insufficiently understood. For example, experiments found that antigen stimulation and co-stimulation at the same time may not be the optimal approach for activation of T cell responses. Pablo Penaloza-MacMaster et al. [103] reported that delayed reinforcement of co-stimulation results in superior CD8 T cell responses relative to early reinforcement of co-stimulation. The study employed an added treatment with co-stimulatory antibodies, which highlights the complexity of the pathways involved in achieving the required co-signaling. Penaloza-MacMaster et al. did not provide reasons why such a codelivery was unsuccessful. They also did not consider that CD8 T cell activation may involve antigen cross-presentation.
The need for effective intracellular adjuvant delivery and release [16,59] The intracellular delivery of adjuvants is essential to bind to intracellular PRRs and trigger subsequent activation of downstream signaling pathways. This is facilitated by advanced vaccine delivery systems such as LNPs, polymer-based carriers, and other nanomaterials. Certain delivery systems fuse with or disrupt endosomal membranes, releasing distinct adjuvants into the cytosol for interaction with cytoplasmic PRRs [59].	These considerations also do not seem to extend to mRNA vaccines to harness antigen cross-presentation and enable specific cellular immunity: Their modus operandi involves a process. The nature and identity of the inherent adjuvants are insufficiently known; especially during antigen cross-presentation, their involvement cannot be clearly targeted. In part, the inherent adjuvants are only released inside a transfected cell. Others, such as the LNP components, may directly be acquired by cross-presenting cells. Professional APCs that acquire material from transfected somatic cells may internalize a host of different immunogenic compounds. It is not known how this dynamic aspect could be controlled to activate innate immune receptors in cross-presenting APCs.

## Data Availability

Not applicable.

## Abbreviations

The following abbreviations are used in this manuscript:

APCs	antigen-presenting cells
BM	bone marrow
CTLs	cytotoxic T lymphocytes
DC	dendritic cell
mDC	migratory DC
dLNs	draining lymph nodes
dsRNA	double-stranded RNA (ribonucleic acid)
ELISpot	enzyme-linked immunosorbent spot assay
EV	extracellular vesicle
IFN	interferon
LN	lymph node
LNP	lipid nanoparticle
MHC	major histocompatibility complex
PAMPs	pathogen-associated molecular patterns
SAMs	self-amplifying mRNA vaccines
TAP	the transporter associated with antigen processing
1-ψ	1-methylpseudouridine
